# Strain prevalence and killer factor only partially influence the fermentation activity of pairwise *Saccharomyces cerevisiae* wine strains inoculation

**DOI:** 10.1371/journal.pone.0300212

**Published:** 2024-04-29

**Authors:** Jacopo Sica, Chiara Vendramini, Chiara Nadai, Zeno Molinelli, Milena Carlot, Alessio Giacomini, Viviana Corich

**Affiliations:** 1 Department of Agronomy Food Natural Resources Animals and Environment (DAFNAE), University of Padova, Legnaro (PD), Italy; 2 Interdepartmental Centre for Research in Viticulture and Enology (CIRVE), University of Padova, Conegliano (TV), Italy; Tulane University Health Sciences Center, UNITED STATES

## Abstract

Commercial *Saccharomyces cerevisiae* starters are single-strain cultures widely used in winemaking to optimise the fermentation process and improve the organoleptic quality of wine. Unfortunately, the worldwide extensive use of a limited number of industrial strains led to the standardisation of the sensory properties, reducing the identity of wines. Therefore, the use of multi-strain *S*. *cerevisiae* starters can be an alternative tool to alter the sensory profile of wines, increasing the diversity of wine styles. However, this strategy may be interesting only if the overall fermentation kinetics is not affected. To date, there is a lack of information regarding the influence of multi-strain starters on the overall fermentation process in wine. In this context, killer toxins, affecting the viability of sensitive strains, can play a significant role. This study aimed to evaluate the effects of pairing eight wine strains of *S*. *cerevisiae* (two sensitive, three neutral and three killer) in co-fermentations compared to single-strain fermentations. Results evidenced that, among co-fermentations where the strain prevalence was significant, the killer strains constituted 79% to 100% of the total yeast population when co-inoculated with a sensitive one. However, in most of the cases, co-fermentations kinetics were similar to those of sensitive strains or worse than both strains. Thus, the presence of a killer strain alone is not sufficient to predict the overall fermentation progress, which is an essential information in winemaking. Interestingly, the neutral strain P304.4 was always prevalent, regardless of the second strain and, in most of the co-fermentations, the overall fermentation trend was similar to the P304.4 single-strain fermentation. Regardless of killer activity, our results suggest that the effect of strains on fermentative kinetics is still unpredictable, and further studies are needed to thoroughly explore strain to strain interactions in winemaking.

## Introduction

*Saccharomyces cerevisiae* is the main agent of alcoholic fermentation and it is widely used as a starter in various fermentation processes, such as those involved in wine, beer and bread production. When grape juice undergoes alcoholic fermentation, *S*. *cerevisiae* becomes the prevalent species, particularly as the ethanol concentration increases [1].

In the winemaking industry, commercial starter cultures of *S*. *cerevisiae* have become commonplace since the development of active dry wine yeasts in the 20th century. These starter cultures allow winemakers to maintain good control over the fermentation process [2]. By using these commercial *S*. *cerevisiae* starters, winemakers can always achieve consistent, replicable, and well-regulated fermentations. However, a consequence of the extensive use of a limited number of commercial yeasts is the standardisation of wines, which leads to a loss of organoleptic complexity, hence flattening wine quality [3].

To counteract the homogenisation of wines and preserve their distinctiveness, some winemakers started looking for some alternatives to perform alcoholic fermentation. One such approach involves using multi-strain starter cultures. Numerous studies have been conducted to examine the effects of employing multi-starters based on mixed or sequential inoculation, using different pour fermenting yeast species together with a technologically relevant *Saccharomyces* strain, mimicking what happened in a spontaneous fermentation [4–10]. The management of non-*Saccharomyces* yeasts in the winery is very challenging due to their diverse nutritional requirements and fermentation performances [11]. Therefore, the use of a multi-starter composed of different strains of *S*. *cerevisiae*, able to improve wine quality, is more easily manageable as their requirements are similar. One of the advantages of this strategy is the contribution to wine regional typicality when indigenous *S*. *cerevisiae* strains are used and the increase of wine complexity [2,12,13]. On the contrary, to date, there is limited available information regarding the co-inoculation of different strains of *S*. *cerevisiae* for wine fermentation [14–17] and even less studies described the effects of the simultaneous presence of multiple *S*. *cerevisiae* strains on the overall fermentation kinetics. Some of these studies have identified chemical differences in the wines produced. Howell et al. [14] observed a strict metabolic interaction among yeast strains, noting that the profiles of wines produced through mixed culture fermentation differed from those fermented in monoculture. These differences could not be replicated by blending wines that were fermented individually.

When employing these multiple starters, several key factors warrant evaluation, including the competition between the strains and their persistence during the fermentation process. Additionally, the killer phenotype of each strain must be considered, as this trait strongly influences the competition ability. Several strains of *S*. *cerevisiae* are referred to as killer yeast (K) due to their ability to produce an extracellular toxin that is lethal to other strains known as sensitive (S) [18]. Additionally, there are neutral (N) yeasts that are resistant to the killer toxin but do not produce it themselves [19]. *S*. *cerevisiae* killer toxins have been grouped into four types (K1, K2, K28 and Klus). Killer type K2 is frequently found in winery environments, as it is the only toxin active at wine pH (3–3.5), giving a selective advantage to killer strain [20]. In fact, K2 toxin activity is assessed between pH 2.8 and 4.8 [21]. Moreover, the killer phenotype is commonly considered an important criterion for the selection of wine yeast starters [22]. In fact, if selected sensitive strains are inoculated into grape juice, they could be inhibited by wild killer yeasts during fermentation. The release of killer toxins has been considered a competitive advantage leading to changes in the growth dynamics of the yeast population. However, the prevalence of *S*. *cerevisiae* killer strains is not predictable and depends on various factors, such as the killer/sensitive ratio at the inoculum. Contradictory results have been reported regarding the adequate initial ratio that allows killer yeasts to predominate fermentation environments. Killer cells, even when inoculated at levels as low as 0.01% of the total *S*. *cerevisiae* yeast population, have been shown to dominate during grape juice fermentation [23]. On the contrary, other authors found the killer prevalence when the killer and sensitive strains were inoculated in equal proportions [18] or when the killer strain was inoculated at proportions higher than 50% [24]. In other studies, a very high killer/sensitive ratio (25/1 or 100/1) was necessary to supplant a sensitive yeast [25,26]. However, the extent of the killer effect in wine fermentation must be influenced by other factors since killer and sensitive yeasts coexist in nature [27]. The presence of neutral strains that can counteract killer predominance [28], the size of the inoculum, the environmental conditions and nitrogen level in the must [19,29], as well as the susceptibility of sensitive strains to the killer toxins [23], have been demonstrated to modulate the killer phenotype. Although the killer activity does not greatly influence industrial fermentations where a single strain is present at high concentrations, when applying the multi-starter strategy using different *S*. *cerevisiae* strains, assessing the impact of the presence of killer, neutral or sensitive strains becomes crucial. Moreover, to our knowledge, no studies have been conducted to date aimed at determining whether the presence of different strains enhances or slows down the overall fermentation kinetics. Hence, the use of novel starter cultures based on multiple *S*. *cerevisiae* strains could prove to be a promising strategy to enhance the complexity of wine flavours. However, these aspects are appealing only if the overall fermentation kinetics remain optimal.

Therefore, the main objective of this study was to assess the impact of pairwise inoculation of eight wine strains of *S*. *cerevisiae*, on the fermentation kinetics with respect to the corresponding single-strain inoculation. Special attention was given to understanding the influence on by-product formation, and the persistence of these yeast strains in the fermentation medium.

## Materials and methods

### Yeast strains

For this study eight indigenous strains, named P283.4, P234.15, P254.12, P301.9, P304.4, P301.4, P138.4 and B173.4, were used. These yeasts were isolated from vineyards in the winemaking area of Prosecco Superiore di Conegliano Valdobbiadene DOCG (Italy) [30]. For the yeasts isolation from vineyard the authors did not require a permit to access the field site as the sampled vineyards belonged to wineries that were partners of that research project (BIODILIEVITI), as well as the Consorzio di Tutela del Prosecco Superiore di Conegliano Valdobbiadene DOCG, and the sampling was planned as part of the project.

### PCR-amplification of inter-delta sequences

Cell suspension for DNA amplification was performed as described by Nadai et al. [31]. Two μl of the cell suspension were used for PCR amplification. Oligonucleotide primers delta12 (TCAACAATGGAATCCCAAC) and delta21 (CATCTTAACACCGTATATGA) were used to amplify total genomic DNA between the repeated interspersed delta sequences, as previously described [32]. PCR products were separated in 1.5% agarose gels in 0.5 × TBE buffer. The molecular marker 100 bp DNA ladder (Promega, Madison, USA) was used as the molecular size standard. Electrophoresis gels were stained with EuroSafe Nucleic Acid Stain (Euroclone, Pero, Italy), and visualised by UV transillumination. Digital images were acquired with an EDAS290 image capturing system (Kodak, Rochester, NY). Clustering of profiles was performed using the GelClust software [33].

### Killer assay

The killer phenotype was tested according to Romano et al. [34]. The tests were performed on YPD medium containing yeast extract (Difco, Milano, Italy) 10 g/l, peptone (Difco, Milano, Italy) 20 g/l, glucose (Difco, Milano, Italy) 20 g/l and agar (Difco, Milano, Italy) 20 g/L, buffered at pH 4.5. Before the plate pouring, a filtered sterilised solution containing Methylene blue was added to the YPD medium to reach a final concentration of 0.003%. An aliquot of 0.1 ml of cell suspension (containing 10^5^ cells/ml) of each potential sensitive strain was spread on YPD Petri dishes. A loopful of cells from 24-hour culture of all the strains (including the potential sensitive) to be tested as potential killer was transferred onto the solid media. Finally, the plates were incubated at 25°C for 4 days. The commercial yeast EC1118 (Lallemand Inc., Montreal, Canada) was used as the reference strain, as it is a well-known killer strain according to the manufacturer declaration. The tested strain was classified as killer if the colony grown on the plate was surrounded by an inhibition halo indicating no growth of the strain whose culture was spread on the plate. The latter strain was classified as sensitive. If no inhibition halo was observed, the tested strain was designated as neutral.

### Fermentation trials and strain prevalence

Pre-cultures of each strain used in this work were prepared as described by Bovo et al. [35]. The concentration of each strain in a stationary phase YPD (yeast extract–peptone–dextrose, Difco, Milan, Italy) culture was determined by flow cytometry count using a CyFlow SL flow cytometer (Partec, Münster, Germany), following the manufacturer’s instructions. Yeast pre-cultures were diluted to an appropriate concentration for flow cytometer counting (approximately 10^5^ cells/ml), and the data were analysed using the FloMax data acquisition and analysis software (Partec, Münster, Germany). Flow cytometry cell count data were used to set the correct cell dilution to reach an inoculum concentration of 1 × 10^6^ cells/ml in single-strain fermentations and 5 × 10^5^ cells/ml, for each strain, in co-fermentations. Fermentations were carried out in 100 ml-capacity Erlenmeyer flasks sealed with a silicone cap and supplied with a bowed glass pipette to enable the CO_2_ to escape. The flasks contained 100 ml of MS300 synthetic must, according to Bely et al. [36]. Fermentation replicates ranged from three to six. The number of replicates was increased when fermentation trends exhibited a wide variability.

All the flasks were kept at 25°C until the end of fermentation. Alcoholic fermentation was monitored by measuring the weight loss, due to CO_2_ production, twice a day throughout the whole fermentation process. Each fermentation was stopped when the weight loss was lower than 0.1 g after 24 h. At the end of fermentation, the supernatant was stored at -20°C for chemical analysis.

To determine strain proportions in co-fermentations, samples were collected when alcohol content reached approximately 6.5%, and colony isolation was performed on YPD agar plates. A total of 12 colonies, randomly chosen from isolation plates within the same dilution series, were submitted to the amplification of inter-delta region. 95% confidence intervals for the proportion of strains were calculated using a normal approximation for a finite population size (i.e. the total number of grown colonies).

### Chemical analysis

HPLC was used to determine the concentrations of residual glucose, glycerol, succinic acid and acetic acid, as described by Nadai et al. [37]. Ten μl of filtered sample were analysed using a Waters 1525 HPLC binary pump (Waters, Milford, MA, USA) equipped with a 300 × 7.8 mm Aminex HPX_87H HPLC column (Bio-Rad, Hercules, CA, USA). A Waters 2414 Refractive Index Detector (Waters, Milford, MA, USA) was used for the determination of glucose, fructose and glycerol, while acetic and succinic acids were determined using a Waters 2487 Dual Absorbance Detector (Waters, Milford, MA, USA) set at 210 nm. The analyses were performed under isocratic conditions at 0.6 ml/min and 65°C using 0.01 N H_2_SO_4_ as the mobile phase. The concentrations, expressed as g/l, were calculated by using calibration curves of the individual compounds, and peak areas were determined using Waters Breeze 2 software (Waters, Milford, MA, USA), an internal software that acquired and processed the data.

### Fermentation kinetics modelling and statistical testing

The kinetic data were used to fit an integrated Gompertz model, as reported for example by Manan and Webb [38]. In this model, the produced CO_2_ is a function of time (*t*) according to Equation [CO_2_] = [CO_2 max_]exp(-*b*exp[-*kt*]), where [CO_2 max_] is the maximum CO_2_ concentration (at *t* –> ∞) (g/l); *b* is a constant related to the initial conditions (dimensionless); *k* is the specific CO_2_ evolution rate (h^-1^); *t* is the fermentation time (h).

To compare the kinetics of the different fermentations, each fermentation replica was individually fitted with a Gompertz model. Subsequently, the following parameters were extracted from each equation: CO_2 max_ (maximum CO_2_ production), CO_2_ evolution rate (*k*), related to the fermentation speed, and time at the inflection point (*t*_*inf*_ = ln(*b*)/*k*), when CO_2_ production rate starts to decrease. These extracted parameters were then subjected to a principal component analysis (PCA) to visualise the differences between the curves. The statistical significance of these differences was assessed using Permutational Multivariate Analysis of Variance (PERMANOVA). Similarly, for the analysis of differences in chemical data among the fermentations, PCA and PERMANOVA were performed. Furthermore, to examine whether differences in kinetic parameters were associated with differences in chemical data collected at the end of fermentation, the Spearman correlation test was applied to pairs of observations and the Mantel correlation test was conducted to compare the distance matrices based on kinetic parameters and chemical data.

The statistical analyses and data visualisation were conducted using the Python libraries scikit-learn [39], statsmodels [40], and matplotlib [41].

## Results and discussion

### Genetic relationship and fermentation behaviour of eight vineyard *S*. *cerevisiae* strains

**Fig 1:**
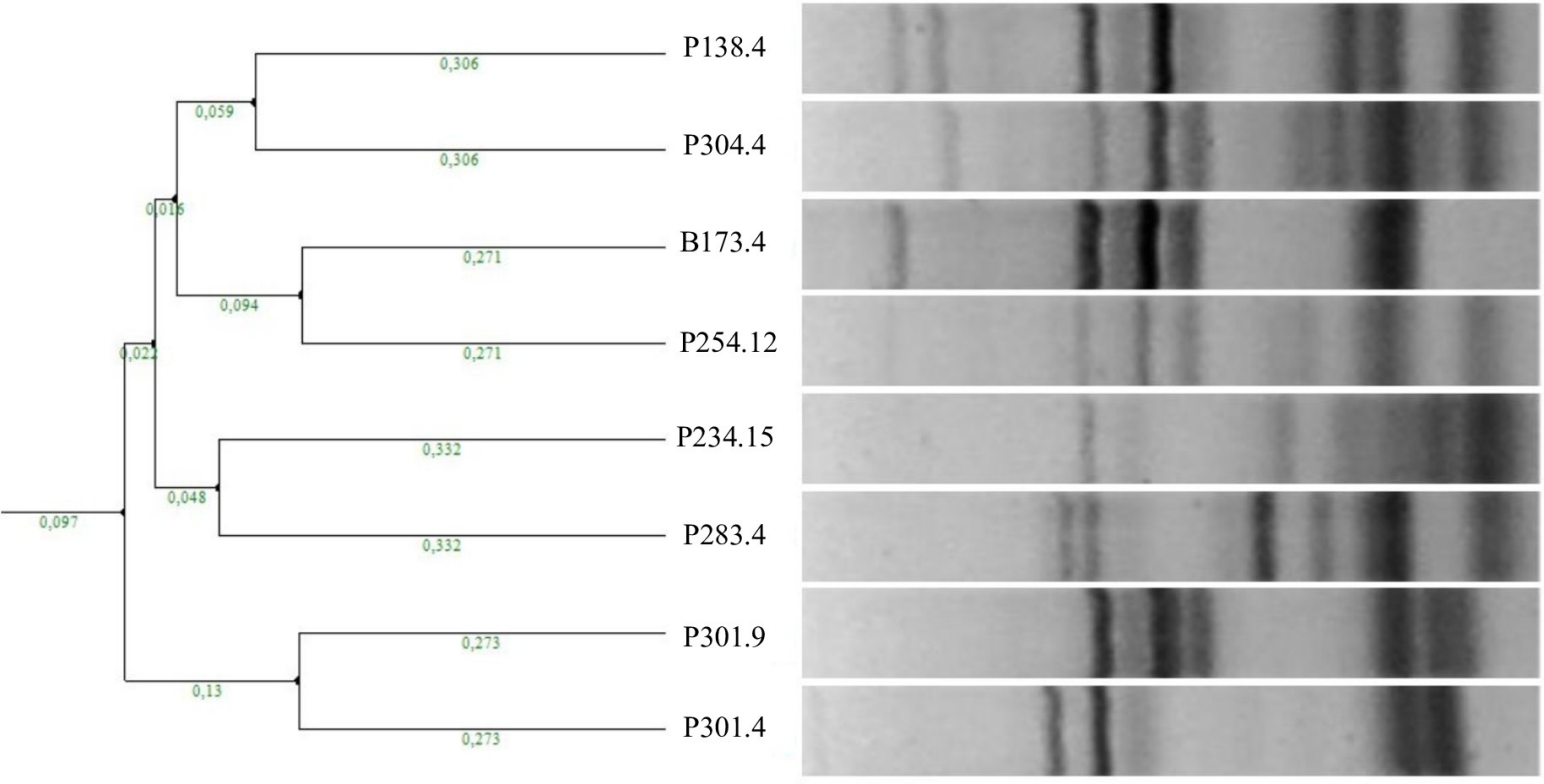
Inter-delta clustering of *S*. *cerevisiae* strains. Jaccard distances are displayed for each branch.

The strains used in this study are part of a large collection of *S*. *cerevisiae* yeasts obtained through the sampling of healthy grape bunches in the vineyard, which were individually crushed and left to ferment. Yeasts were isolated from the partially spontaneously fermented juice of Glera grapes and identified based on their genetic profiles [30]. The sampling area allowed for the identification of 37 strains, and 8 strains were selected as representative: P283.4, P234.15, P254.12, P301.9, P304.4, P301.4, P138.4, B173.4. Their genetic profiles, obtained through inter-delta analysis, are reported in **Fig 1**. Two main clusters, based on similarity, are present: the first group included strains P301.4 and P301.9, while the other comprised the remaining strains. Interestingly, the two strains in the first group (P301.4 and P301.9) were isolated from the same grape bunch.

The strains were used to ferment synthetic must MS300 mimicking the oenological environment. A total of 8 single-strain fermentations and 28 co-fermentations were performed.

To assess the impact of co-presence of two strains on individual strain fermentation patterns, single-strain fermentations were first carried out, and their kinetics and secondary products were measured (**S1 Fig and S1 Table**).

**Fig 2:**
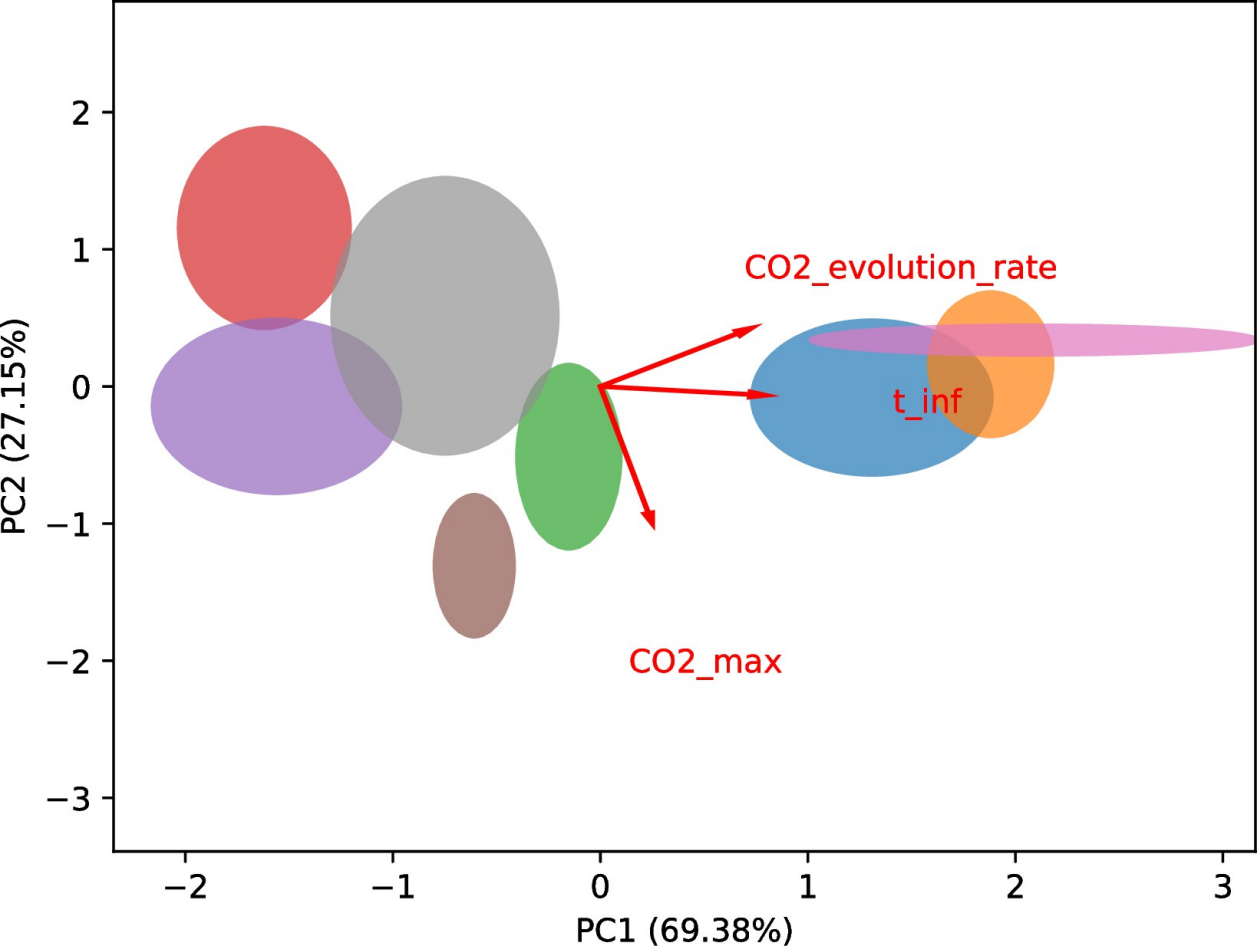
Principal component analysis (PCA) biplot of Gompertz model-derived kinetic parameters (time at inflection point (*t*_*inf*_), CO_2_ evolution rate and CO_2 max_) obtained from single-strain fermentations. The arrows represent scaled variable loadings and the ellipses represent the variability in terms of standard deviation of the principal components among the replicates. ■ B173.4, ■ P138.4, ■ P234.15, ■ P254.12, ■ P283.4, ■ P301.4, ■ P301.9, ■ P304.4. PC1: Principal component 1; PC2: Principal component 2.

To evaluate differences between fermentation trends, the weight loss data due to CO_2_ production were used to fit the Gompertz model, which proved to be effective in describing the data (overall R-squared of 0.99). Model fitting allowed to estimate three kinetic parameters that are useful to describe the fermentation process: the maximum amount of CO_2_ produced (CO_2 max_) at the end of the process; the CO_2_ evolution rate, which is proportional to the fermentation speed; the time at the inflection point (*t*_*inf*_), that indicates the moment when the rate of CO_2_ production reaches its maximum value before starting to decrease. Among the analysed fermentation processes, those in which CO_2 max_ got closer to the theoretical value based on the initial sugar concentration, CO_2_ evolution rate increased and *t*_*inf*_ decreased, resulted in better fermentation kinetics. These parameters, estimated for each fermentation replica (ranging from three to six), are shown in **Table 1**, and were subsequently subjected to a PCA to compare the overall trends of the fermentations (**Fig 2**).

**Table 1:** Gompertz model-derived kinetic parameters obtained from single-strain fermentations.

Strain	CO_2 max_ (g/l)	CO_2_ evolution rate (h^-1^)	*t*_*inf*_ (h)
**B173.4**	92.54 ± 2.77	0.026 ± 0.005	43.75 ± 4.32
**P138.4**	92.44 ± 2.20	0.031 ± 0.004	39.49 ± 2.99
**P234.15**	91.65 ± 3.12	0.018 ± 0.002	61.47 ± 6.99
**P254.12**	80.40 ± 4.49	0.014 ± 0.001	70.47 ± 2.64
**P283.4**	86.77 ± 2.54	0.014 ± 0.003	81.49 ± 11.28
**P301.4**	94.57 ± 2.72	0.015 ± 0.001	72.21 ± 3.82
**P301.9**	91.92 ± 2.15	0.034 ± 0.008	39.44 ± 7.68
**P304.4**	85.40 ± 5.01	0.016 ± 0.002	62.10 ± 10.18

CO_2 max_: Maximum CO_2_ produced; *t*_*inf*_: Time at inflection point.

The time at inflection point ranged from 39.44 ± 7.68 to 81.49 ± 11.28 hours, while maximum CO_2_ production ranged from 80.40 ± 4.49 to 94.57 ± 2.72 (g/l). Additionally, the CO_2_ evolution rate ranged from 0.01 to 0.03 ± 0.01 (h^-1^).

PC1 was responsible for 69.38% of the variation in the data and it is mainly related to the at inflection point coefficient and CO_2_ evolution rate. PC2 covered 27.15% of the variance, including mainly maximum CO_2_ production.

The fermentation activity was different among the strains. The best performing strains were B173.4, P138.4, P301.9 that showed the highest fermentation rate throughout the fermentation process (CO_2_ evolution rate) and a decrease in the time at the inflection point (*t*_*inf*_) that occurs earlier than the rest of the strains. Interestingly, strain P301.4, although slower than the best performing group, produced a high amount of CO_2_. A pairwise comparison was made considering the fermentation parameters of each single-strain fermentation kinetics (**Table 2**). The group of best performing strains (B173.4, P301.9, P138.4) showed non-significant differences in the fermentation trends within the group. Moreover, no significant differences were found in the pairs P304.4 - P.234.15, P301.4—P234.15, P283.4—P254.12 and P304.4—P254.12.

**Table 2:** Pairwise comparison between fermentation kinetics.

	B173.4	P138.4	P234.15	P254.12	P283.4	P301.4	P301.9	P304.4
**B173.4**								
**P138.4**	*p* = 0.207							
**P234.15**	*p* = 0.009*	*p* = 0.023*						
**P254.12**	*p* = 0.008*	*p* = 0.031*	*p* = 0.031*					
**P283.4**	*p* = 0.008*	*p* = 0.012*	*p* = 0.048*	*p* = 0.083				
**P301.4**	*p* = 0.01*	*p* = 0.013*	*p* = 0.04*	*p* = 0.007*	*p* = 0.063			
**P301.9**	*p* = 0.363	*p* = 0.985	*p* = 0.027*	*p* = 0.029*	*p* = 0.011*	*p* = 0.006*		
**P304.4**	*p* = 0.007*	*p* = 0.013*	*p* = 0.435	*p* = 0.106	*p* = 0.021*	*p* = 0.027*	*p* = 0.006*	

Results of the PERMANOVA tests, based on the CO_2 max_, CO_2_ evolution rate, and *t*_*inf*_ parameters. *p*-values < 0.05 indicate significant differences between the fermentation kinetics of different strains and are marked with an asterisk.

**Fig 3:**
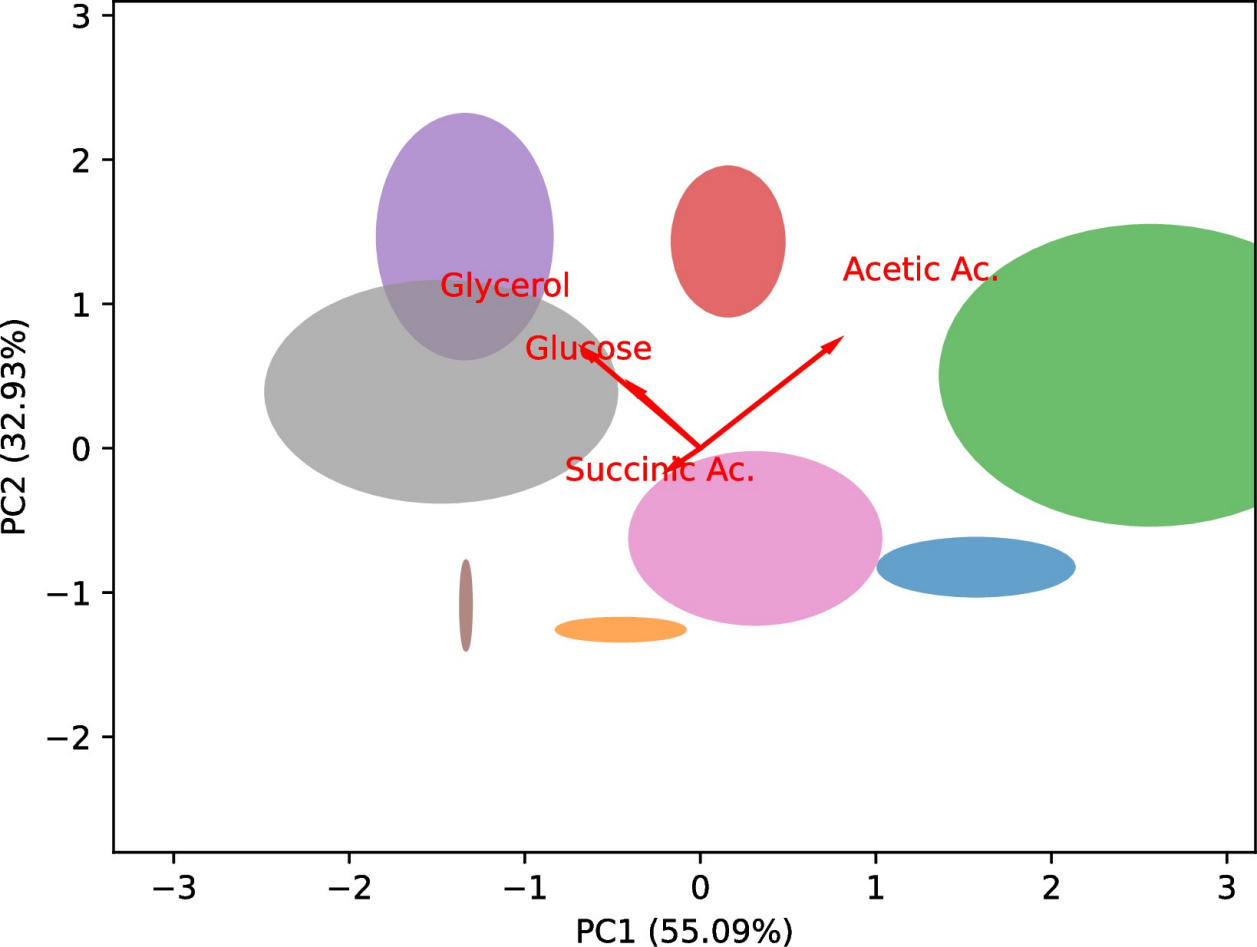
Principal component analysis (PCA) biplot of secondary products (glucose residues, glycerol, succinic and acetic acids) at the end of single-strain fermentations. The arrows represent scaled variable loadings and the ellipses represent the variability in terms of standard deviation of the principal components among the replicates. ■ B173.4, ■ P138.4, ■ P234.15, ■ P254.12, ■ P283.4, ■ P301.4, ■ P301.9, ■ P304.4. PC1: Principal component 1; PC2: Principal component 2.

At the end of fermentation, glucose residues and glycerol, succinic and acetic acids were measured (**S1 Table**) and Principal Component Analysis (PCA) analysis was used to interpret differences among the levels of these fermentation products (**Fig 3**). These metabolites are important from a technological perspective as they allow the assessment of the regularity of the fermentation process (glucose residues and succinic acid) and the quality of the final product (glycerol and acetic acid).

The measurement of sugar residues, along with fermentation time, can be utilised to assess the performance of strain fermentation. Typically, a longer fermentation time results in higher glucose residue levels. In single-strain fermentations, the glucose residue range among the strains was wide, from 0.58 ± 0.36 to 11.46 ± 3.52 g/l. Glycerol is the predominant secondary compound in alcoholic fermentation. It imparts structure and smoothness to the wine, while also playing a crucial role in defining its flavour and aroma profile. Yeasts produce glycerol at the onset of fermentation as a survival mechanism in response to high sugar concentrations and osmotic stress [21]. The concentration of glycerol varies depending on the yeast strains. The glycerol concentrations generated at the conclusion of single-strain fermentations ranged between 4.13 ± 0.14 and 5.45 ± 0.33 g/l. Among the strains, B173.4 and P234.15 exhibited higher glycerol production, whereas strain P304.4 yielded a lower glycerol concentration. In the initial stages of fermentation, the surplus pyruvate generated through glycolysis combines with CO_2_ using the biotin-dependent enzyme pyruvate carboxylase, resulting in the production of oxaloacetate. Subsequently, oxaloacetate undergoes transformation into succinate [21]. Therefore, its concentration level is due to the strain’s ability to set up the alcoholic fermentation. Strains of *S*. *cerevisiae* are recognized for generating succinic acid at levels of 200 mg/l to 2 g/l [42]. In this work, the strain’s production of succinic acid during single-strain fermentation fell within the range of 0.19 ± 0.03 to 0.38 ± 0.04 g/l. Acetic acid is generated by yeast during alcoholic fermentation, through the oxidation of acetaldehyde and used to synthesise fatty acids and sterols [21]. When the concentration exceeds 0.7 g/l, the volatile acidity becomes noticeable and affects the wine’s quality by intensifying its harshness and astringency [43]. The average acetic acid production values at the completion of fermentation by the individual strains ranged from 0.38 ± 0.04 to 0.69 ± 0.16 g/l, and lower than the detection threshold. Notably, strains P254.12 and P234.15 exhibited the highest acetic acid production, while strains P301.4 and P138.4 demonstrated the lowest production.

The Mantel correlation test was conducted to compare the distance matrices based on kinetic parameters and chemical data. The overall correlation was weak (~0.30), but significant (*p*<0.01) and it was primarily driven by glucose residue, whose distance matrix alone was found to be significantly (*p*<0.01) correlated with the distance matrix based on kinetic parameters by ~0.29. Actually, residual glucose at the end of fermentation was significantly (*p*<0.01) negatively correlated with CO_2_ evolution rate (-0.61) and CO_2_ max (-0.65) and positively correlated with time at inflection point (0.58).

The reported data indicates that, despite differences among strains, the analysed yeasts exhibited good fermentative behaviour, entirely comparable to that of commercial wine starters, and a production of secondary metabolites whose concentrations fell within the oenological standard.

### Killer phenotype

One of the most well-known mechanisms capable of modulating interactions between yeasts present in grape must is the production of killer toxins. The killer phenotype undoubtedly enhances competitiveness, favouring killer (K) strains over sensitive (S) strains, but not over neutral (N) strains, which are non-producing and insensitive. When selected sensitive yeasts are inoculated into the must, they may be suppressed by wild killer yeasts during fermentation. Therefore, commercial strains are generally of the killer type. In the killer test the commercial yeast EC1118 was used as the killer reference strain. The killer activity of the eight strains, as well as the reference strain, was determined through a cross test, where each strain was examined as a potential producer and subsequently as a potential sensitive strain. Killer strains were identified as those capable of inhibiting the growth of at least one strain (referred to as sensitive), resulting in the formation of an inhibition halo. Neutral strains, on the other hand, were defined as strains that, despite lacking killer activity, were able to grow on the plate when in contact with a killer strain, without exhibiting an inhibition halo. Results (**S2 Fig**) indicated the presence of three killer strains (P283.4, P254.12, and B173.4), two sensitive strains (P301.4 and P301.9), and three neutral strains (P138.4, P234.15, and P304.4). Among the tested strains, the two sensitive strains proved to be susceptible to all killer strains, while each killer strain exhibited resistance to all other killer strains. Interestingly, the two sensitive strains were those that clustered together based on similarity (**Fig 1**) and were isolated from the same grape bunch.

### Co-fermentations and strain prevalence

The fermentation kinetic parameters (*t*_*inf*_, CO_2_ evolution rate and CO_2 max_) obtained using the Gompertz model for each co-fermentation replica (ranging from three to six), provided in **Table 3**, were compared to the values obtained from the respective single-strain fermentations (**Table 1**), with PERMANOVA tests.

**Table 3:** Gompertz model-derived kinetic parameters obtained from co-fermentations.

Strains	CO_2 max_ (g/l)	CO_2_ evolution rate (h^-1^)	*t*_*inf*_ (h)
**B173.4-**P138.4	89.12 ± 4.31	0.018 ± 0.004	59.24 ± 11.15
**B173.4-**P234.15	96.33 ± 6.86	0.016 ± 0.001	64.71 ± 8.70
**B173.4-P254.12**	89.82 ± 2.55	0.018 ± 0.002	60.80 ± 6.27
**B173.4-***P301*.*4*	87.47 ± 4.48	0.016 ± 0.001	65.51 ± 4.10
**B173.4-***P301*.*9*	91.85 ± 2.41	0.025 ± 0.002	49.47 ± 3.27
P138.4**-P254.12**	91.48 ± 0.84	0.017 ± 0.003	64.44 ± 8.01
P138.4**-***P301*.*4*	92.65 ± 1.52	0.019 ± 0.005	61.46 ± 9.93
P138.4**-***P301*.*9*	90.60 ± 3.18	0.023 ± 0.009	53.07 ± 7.85
P234.15-P138.4	93.74 ± 3.55	0.026 ± 0.006	47.04 ± 11.08
P234.15**-P254.12**	93.25 ± 1.24	0.030 ± 0.015	48.52 ± 6.70
P234.15**-***P301*.*4*	97.11 ± 0.85	0.018 ± 0.004	59.49 ± 11.90
P234.15**-***P301*.*9*	93.59 ± 2.44	0.041 ± 0.001	36.05 ± 5.56
**P283.4-B173.4**	91.06 ± 4.15	0.022 ± 0.003	49.09 ± 6.50
**P283.4-**P138.4	94.53 ± 2.19	0.015 ± 0.001	71.91 ± 7.04
**P283.4-**P234.15	88.93 ± 1.72	0.012 ± 0.001	86.50 ± 9.25
**P283.4-P254.12**	85.88 ± 2.43	0.014 ± 0.001	77.67 ± 6.08
**P283.4-***P301*.*4*	88.50 ± 4.50	0.013 ± 0.001	91.03 ± 6.48
**P283.4-***P301*.*9*	92.05 ± 4.78	0.017 ± 0.001	69.52 ± 5.53
**P283.4-**P304.4	82.23 ± 4.59	0.010 ± 0.001	102.41 ± 12.73
*P301*.*4***-P254.12**	90.41 ± 2.13	0.015 ± 0.001	67.69 ± 6.94
*P301*.*9***-P254.12**	96.12 ± 4.46	0.014 ± 0.002	78.66 ± 13.27
*P301*.*9*-*P301*.*4*	90.95 ± 3.18	0.019 ± 0.005	59.47 ± 9.79
P304.4**-B173.4**	88.53 ± 2.61	0.015 ± 0.001	62.75 ± 4.20
P304.4-P138.4	89.82 ± 3.34	0.014 ± 0.002	72.98 ± 17.60
P304.4-P234.15	86.63 ± 2.83	0.014 ± 0.001	63.76 ± 5.78
P304.4**-P254.12**	87.95 ± 3.13	0.013 ± 0.001	81.70 ± 6.78
P304.4-*P301*.*4*	81.08 ± 8.39	0.013 ± 0.002	73.84 ± 8.60
P304.4-*P301*.*9*	87.48 ± 4.29	0.013 ± 0.002	72.72 ± 11.23

CO_2 max_: Maximum CO_2_ produced; *t*_*inf*_: Time at inflection point. Killer strains are indicated in bold and sensitive strains in italics.

**Fig 4:**
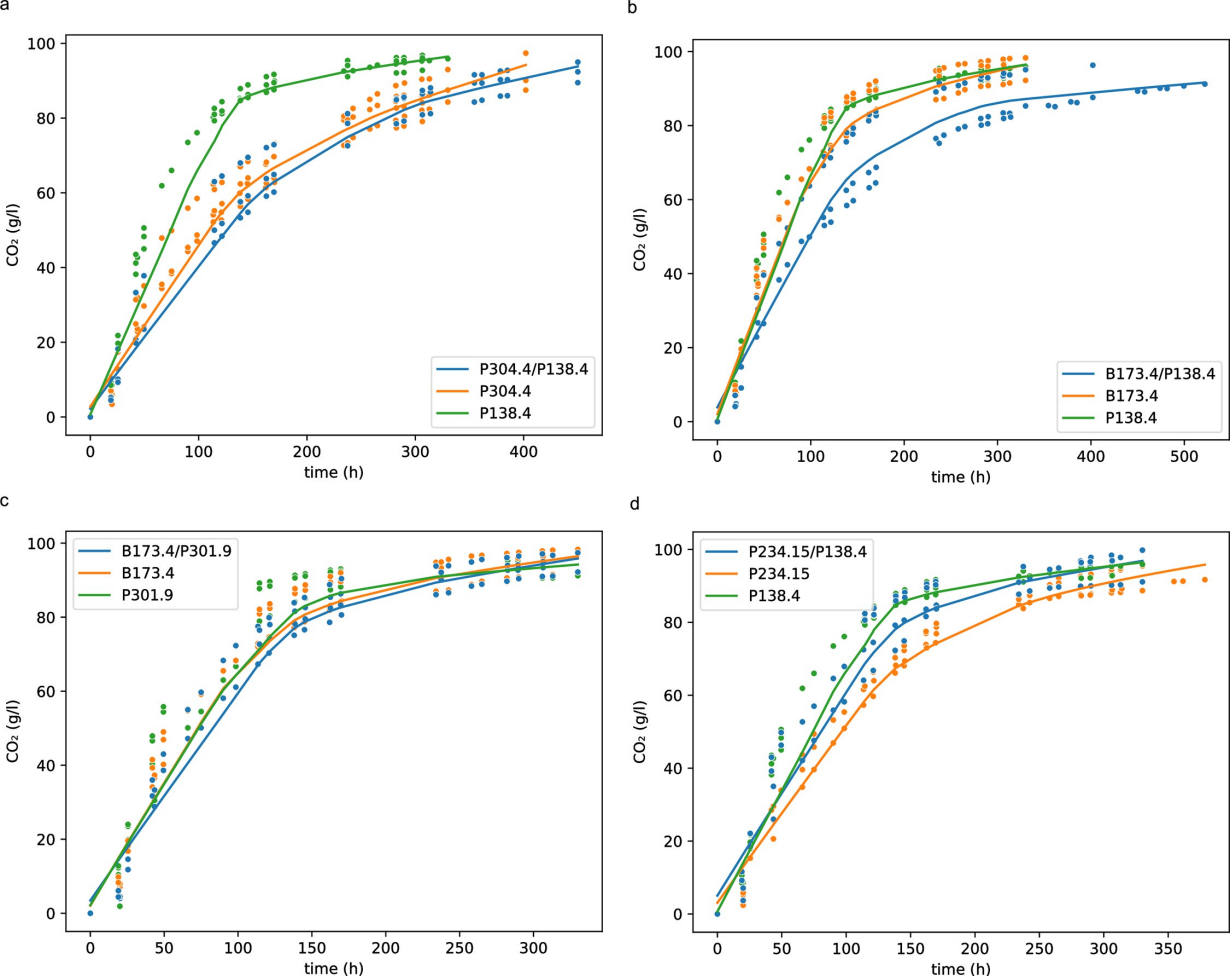
Fermentation kinetics (CO_2_ released/time) in synthetic must. Each graph shows the kinetics trend of two single-strain fermentation kinetics (indicated by the strain name) and their respective co-fermentation kinetics (indicated by the strain names separated by a slash). a) Co-fermentation kinetics classified as “P304.4 like”; b) Co-fermentation kinetics classified as “different”; c) Co-fermentation kinetics classified as “indistinguishable”; d) Co-fermentation kinetics classified as “variable”.

The results of these tests were used to classify the co-fermentation kinetics as follows: “*strain name*_like” if co-fermentation kinetics significantly overlapped with one of the two single-strain kinetics; “different” if co-fermentation kinetics were distinguishable from both single-strain kinetics; “indistinguishable” if all the kinetics (the co-fermentation and the two single-strain) overlapped; “variable” if the two single-strain kinetics were statistically different, but the co-fermentation kinetics were variable among replicas and overlapped with both single-strain kinetics. Examples of co-fermentation kinetics, which were classified in different ways, are provided in **Fig 4**.

The outcomes of PERMANOVA tests and the subsequent classification of all co-fermentations performed are displayed in **Table 4**.

**Table 4:** Classification of co-fermentations in relation to the single-strains fermentation kinetics.

Strain 1–Strain 2	Strain 1 vs Strain 2	Strain 1 vs co-fermentation	Strain 2 vs co-fermentation	Strain 1 and Strain 2 vs co-fermentation	Classification of co-fermentation
**B173.4**-P138.4	*p* = 0.207	-	-	*p* = 0.001*	different
**B173.4**-P234.15	*p* = 0.009*	*p* = 0.02*	*p* = 0.509	-	P234.15_like
**B173.4**-**P254.12**	*p* = 0.008*	*p* = 0.015*	*p* = 0.033*	-	different
**B173.4**-**P283.4**	*p* = 0.008 *	*p* = 0.23	*p* = 0.009*	-	B173.4_like
**B173.4**-*P301*.*4*	*p* = 0.01*	*p* = 0.008*	*p* = 0.011*	-	different
**B173.4**-*P301*.*9*	*p* = 0.363	-	-	*p* = 0.095	indistinguishable
**B173.4**-P304.4	*p* = 0.007*	*p* = 0.02*	*p* = 0.786	-	P304.4_like
P138.4-P234.15	*p* = 0.023*	*p* = 0.285	*p* = 0.106	-	variable
P138.4-**P254.12**	*p* = 0.031*	*p* = 0.03*	*p* = 0.028*	-	different
P138.4-**P283.4**	*p* = 0.012*	*p* = 0.024*	*p* = 0.136	-	P283.4_like
P138.4-*P301*.*4*	*p* = 0.013*	*p* = 0.028	*p* = 0.037*	-	different
P138.4-*P301*.*9*	*p* = 0.985	-	-	*p* = 0.023*	different
P138.4-P304.4	*p* = 0.013*	*p* = 0.044*	*p* = 0.261	-	P304.4_like
P234.15-**P254.12**	*p* = 0.031*	*p* = 0.033*	*p* = 0.016*	-	different
P234.15-**P283.4**	*p* = 0.048*	*p* = 0.025*	*p* = 0.482	-	P283.4_like
P234.15-*P301*.*4*	*p* = 0.04*	*p* = 0.479	*p* = 0.059	-	variable
P234.15-*P301*.*9*	*p* = 0.027*	*p* = 0.04*	*p* = 0.551	-	P301.9_like
P234.15-P304.4	*p* = 0.435	-	-	*p* = 0.647	indistinguishable
**P254.12**-**P283.4**	*p* = 0.083	-	-	*p* = 0.105	indistinguishable
**P254.12**-*P301*.*4*	*p* = 0.007*	*p* = 0.029*	*p* = 0.184	-	P301.4_like
**P254.12**-*P301*.*9*	*p* = 0.029*	*p* = 0.028*	*p* = 0.024*	-	different
**P254.12**-P304.4	*p* = 0.106	-	-	*p* = 0.001*	different
**P283.4**-*P301*.*4*	*p* = 0.063	-	-	*p* = 0.007*	different
**P283.4**-*P301*.*9*	*p* = 0.011*	*p* = 0.147	*p* = 0.031*	-	P283.4_like
**P283.4**-*P304*.*4*	*p* = 0.021*	*p* = 0.092	*p* = 0.008*	-	P283.4_like
*P301*.*4*-*P301*.*9*	*p* = 0.006*	*p* = 0.039*	*p* = 0.021*	-	different
*P301*.*4*-P304.4	*p* = 0.027*	*p* = 0.014*	*p* = 0.158	-	P304.4_like
*P301*.*9*-P304.4	*p* = 0.006*	*p* = 0.008*	*p* = 0.116	-	P304.4_like

Results of PERMANOVA test, based on the CO_2 max_, CO_2_ evolution rate, and *t*_*inf*_ parameters, which was used to compare the kinetics between each pair of single-strain fermentations (Strain 1 vs Strain 2) and the kinetics between the co-fermentation and the two respective single-strain fermentations, separately if the single-strain fermentations were significantly different (Strain 1 vs co-fermentation and Strain 2 vs co-fermentation) or together if not (Strain 1 and Strain 2 vs co-fermentation). The resulting classification of co-fermentation kinetics is shown in the last column. *p*-values < 0.05 indicate significant differences and are marked with an asterisk. Killer strains are indicated in bold and sensitive strains in italics.

In 12 out of 28 combinations, the co-fermentations kinetics overlapped with one of the two respective single-strain fermentations (referred to as “*strain name*_like”), suggesting that one of the two strains carried out the fermentation following its typical pattern, despite the presence of the other strain. In 11 combinations, the co-fermentations kinetics differed from both single-strain fermentations (referred to as “different”), showing the presence of an interaction effect. In 3 cases, all the fermentation kinetics of the two single-strains and co-fermentation overlapped (referred to as “indistinguishable”). Therefore, it was not possible to determine whether either of the two strains conducted the fermentation. In 2 cases, the kinetics of the two single-strain fermentations were statistically different, but the co-fermentation kinetics were variable among replicas and overlapped with both single-strain kinetics (referred to as “variable”).

In conclusion, the overall results showed that, in 11 out of 28 cases, the simultaneous presence of two strains in the co-fermentation led to distinct fermentation performances compared to those of the respective single-strain fermentations.

Secondary products were measured at the end of the co-fermentations (**S2 Table**). Glucose residues ranged from 0.22 ± 0.13 to 24.56 ± 4.46 g/l. The glycerol concentrations generated at the end of the fermentations ranged from 3.67 ± 0.37 to 5.69 ± 0.23 g/l. Succinic acid and acetic acid productions ranged from 0.18 ± 0.03 to 0.43 ± 0.02 g/l and from 0.34 ± 0.07 to 0.76 ± 0.08 g/l, respectively.

The comparison between single-strain and co-fermentations was performed also for secondary metabolites and it showed no significant results.

In order to better understand the effect of the simultaneous presence of both strains on the fermentation kinetics, fermenting synthetic musts were sampled when alcohol reached 6.5%, 12 colonies were randomly chosen and the frequency of the two strains was estimated by comparing the inter-delta profile of each isolate. The 95% confidence intervals for the proportion between the strains were calculated (**Table 5**).

**Table 5:** Strains proportion in co-fermentations.

Strain1–Strain 2	Estimated proportion of Strain 1	Colonies of Strain 1 (out of 12)	Classification of co-fermentation
**P254.12**-*P301*.*4*	79% - 100%	11	P301.4_like
*P301*.*4*-P304.4	0%	0	P304.4_like
**B173.4**-P304.4	0%	0	P304.4_like
P138.4-P304.4	0%	0	P304.4_like
P234.15-*P301*.*9*	77% - 100%	11	P301.9_like
**P283.4**-*P301*.*9*	100%	12	P283.4 like
**P283.4**-P304.4	0%	0	P283.4 like
*P301*.*4*-*P301*.*9*	0%	0	different
**P283.4**-*P301*.*4*	100%	12	different
**B173.4**-*P301*.*4*	100%	12	different
**P254.12**-P304.4	0%	0	different
**B173.4**-*P301*.*9*	100%	12	indistinguishable

Estimated proportion of each strain with respect to the other in co-fermentations. In the cases where both strains were detected through sampling, the 95% confidence interval for the proportion is reported. Only informative confidence intervals, narrower than 23%, are shown. Killer strains are indicated in bold and sensitive strains in italics.

Based on the number of colonies on the isolation plates, the confidence interval has been calculated to statistically assess the percentage of the presence of strains. In 12 out of 28 cases, one of the two strains was prevalent (**Table 5**), with a percentage higher than 77%, while in the remaining cases, due to the interval width, it was not possible to accurately establish strain distribution (**S3 Table**).

When co-inoculated with one of the two sensitive strains, the three killer strains were always prevalent, except for the co-fermentation P301.9 (S)—P254.12 (K). Among the killer strains, P254.12 (K) proved to be less aggressive than the others.

Regarding the comparison of fermentation kinetics, in seven out of 12 cases, co-fermentations exhibited “strain-like” behaviour. Two of these co-fermentations involved strain P301.4 (S). In the co-fermentation P304.4 (N)—P301.4 (S), strain P301.4 was not detected, and the co-fermentation kinetics was similar to P304.4 single-strain fermentation, as Gompertz parameters overlapped. Strain P304.4 single-strain fermentation showed a worse trend than P301.4. In P301.4 (S)—P254.12 (K) co-fermentation, strain P301.4 was present at a percentage lower than 21%. Surprisingly, even if P301.4 was co-inoculated with a killer strain, the kinetics of the co-fermentation was like the P301.4 single-strain fermentation, which showed a better trend than P254.12.

In 4 out of 7 “strain-like” co-fermentations, strain P304.4 (N) was involved, and it consistently prevailed (100%), even when co-inoculated with a killer strain (P283.4). When P304.4 was co-inoculated with sensitive or neutral strains, the co-fermentation kinetics were similar to P304.4 single-strain fermentation, which always showed a worse trend compared to that of the second strain. Surprisingly, when P304.4 was co-inoculated with strain P283.4 (K), the co-fermentation kinetic was similar to P283.4 single-strain fermentation, which showed a worse trend than that of P304.4. Other authors found a neutral strain that was able to prevail during fermentation when co-inoculated with a killer strain [17], but the effect of the presence of the neural strain on fermentation kinetics was not investigated.

Strain P301.9 (S) was involved in 2 out of 7 cases, and it was never prevalent. When P301.9 was co-inoculated with a neutral strain (P234.15), the co-fermentation kinetics was similar to P301.9 single-strain fermentation, which showed a better trend than that of P234.15. When P301.9 was co-inoculated with a killer strain (P283.4), the co-fermentation kinetic was similar to that of the killer strain, showing a worse trend than P301.9.

Four out of 12 co-fermentations resulted in kinetics different from those of the two single-strains. Among these four, three co-fermentations involved strain P301.4 (S) that was never prevalent. When it was co-inoculated with another sensitive strain (P301.9), the co-fermentation kinetics was intermediate with respect to the two single-strain fermentations. When P301.4 was co-inoculated with a killer strain (P283.4 and B173.4), the co-fermentations kinetics were worse than both single strain fermentations. In the co-fermentation involving P304.4 (N)—P254.12 (K), the prevalent strain was the neutral one, and the co-fermentation kinetics was intermediate with respect to the two single-strain fermentations.

Finally, in the co-fermentation with strains B173.4 (K)—P301.9 (S) the kinetics of the single-strain fermentations and of the co-fermentation overlapped, and the prevalent strain was the killer one.

Considering co-fermentations in which a killer strain was co-inoculated with a sensitive one, in all cases, the killer strain was prevalent, as expected. However, only in the case of P283.4 (K)—P301.9 (S), the co-fermentation kinetics was similar to that of P283.4 single-strain fermentation, that showed a worse trend than that of P301.9. In the other cases, co-fermentations kinetics were similar to those of sensitive strains or worse than that of both strains. Considering killer strain prevalence, our results confirmed those of the literature [18]. However, there is no literature evaluating the effect of the simultaneous presence of killer and sensitive strains on the overall fermentation kinetics. Our results indicated that the presence of a killer strain is not sufficient to predict the overall fermentation progress, that is an essential information in winemaking.

As previously reported, various factors, including environmental conditions, can influence the implantation capacity of a yeast starter. In the present study, strain prevalence and fermentation performances were evaluated under standard laboratory-scale conditions, starting from the same inoculum size. Therefore, the prevalence of the strains cannot be attributed to variations in must composition or the inoculum size.

Interestingly, the neutral strain P304.4 was always prevalent, regardless of the second strain, and, in three out of five cases (against a killer, a sensitive, and a neutral strain), it originated a P304.4 like co-fermentation kinetics. These results suggested that other genetic and metabolic factors influence both the prevalence of the strain and the kinetics of co-fermentation. Therefore, in the design of multi-strain starters, it is not possible to predict the behaviour of individual strains, but experimental testing is necessary.

## Conclusions

In modern winemaking, the simultaneous inoculation of multiple yeast strains is proposed to obtain a wine with more complex flavours. However, these aspects are interesting only if the overall fermentation kinetics remains optimal.

In our experiment, generally, we did not observe a synergistic effect due to the presence of the two strains, resulting in better co-fermentation kinetics compared to their respective single-strain fermentations. Moreover, the comparison between single and co-fermentation secondary products showed no significant results.

Among the examined strains, there were strains that produced killer toxins, as well as strains that were sensitive or neutral. We noticed that, although the killer strains were always prevalent when co-inoculated with a sensitive strain, the overall kinetics in some cases resembled that of the sensitive one, even when its kinetics were worse than that of the other strain. These results suggest that interactions during the early stages of development, when one of the two strains is not yet prevalent, can strongly influence the subsequent fermentation process. At this stage, nutrients availability in the must is still very high, therefore, nutrients competition can be excluded, although variations in strain assimilation rates could lead to different strain growth. Other aspects can be involved, such as strain fitness (resistance to ethanol or other metabolites) and cell-to-cell contact and aggregation, mediated by cell surface proteins [44].

In general, regardless of the killer factor, the presence of a prevalent strain with good fermentation kinetics does not guarantee the achievement of equally good fermentation kinetics when co-inoculated with a less performing strain. Finally, surprisingly, the most aggressive behaviour was demonstrated by a neutral strain, confirming that other interaction factors different from killer toxins are involved in strain competition. An investigation aimed at assessing the dynamics between strains in the early stages of fermentation, during which prevalence relationships are established, could provide further insights to fully understand the role of the killer phenotype.

The obtained results highlight the need for further and in-depth studies to understand the key factors affecting interaction between strains in wine fermentation.

## Supporting information

S1 Fig Fermentation kinetics (CO_2_ released/time) in synthetic must, performed with the eight strains employed in this study. (PDF)

S2 Fig Killer and sensitive phenotype assays on agar plates. The name of the strain tested for sensitivity (i.e the strain inoculated in the whole medium) is indicated in the upper left corner of each picture.
(PDF)

S3 Fig Comparison of co-fermentations and single-strain fermentations kinetics (CO_2_ released/time) in synthetic must. Each graph shows the kinetics trend of two single-strain fermentation kinetics (indicated by the strain name) and their respective co-fermentation kinetics (indicated by the strain names separated by a slash).
(PDF)

S1 Table Glucose residues and glycerol, succinic and acetic acids produced at the end of single-strain fermentations. (DOCX)

S2 Table Glucose residues and glycerol, succinic and acetic acids produced at the end of co-fermentations. (DOCX)

S3 Table Estimated proportion between strains in co-fermentations. The total number of grown colonies, the number of sampled colonies for each strain (out of a total of 12 sampled colonies) and the 95% confidence interval for the proportion are reported. Killer strains are indicated in bold and sensitive strains in italics.
(DOCX)
