# Supplementary figures and images for: Strain prevalence and killer factor only partially influence the fermentation activity of pairwise Saccharomyces cerevisiae wine strains inoculation
Source: PLoS One. 2024 Apr 29;19(4):e0300212. doi: 10.1371/journal.pone.0300212 (PMC11057759; doi:10.1371/journal.pone.0300212)

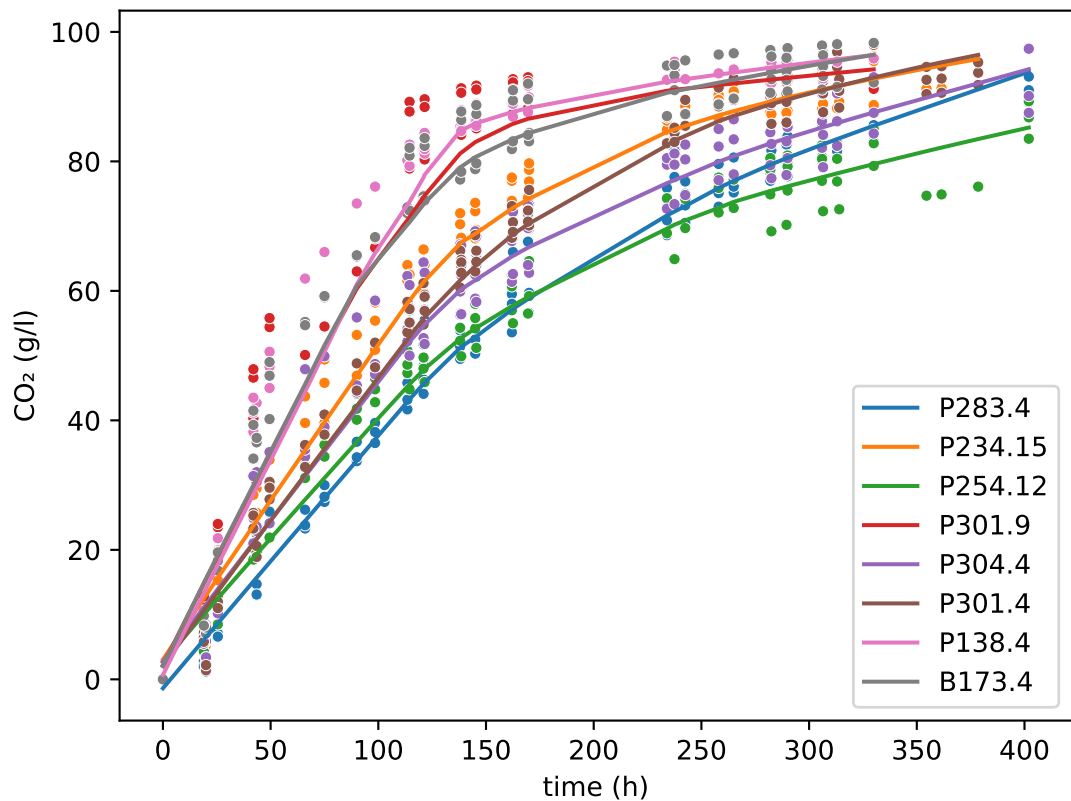

Supplement: S1 Fig — (PDF) [file pone.0300212.s001.pdf]

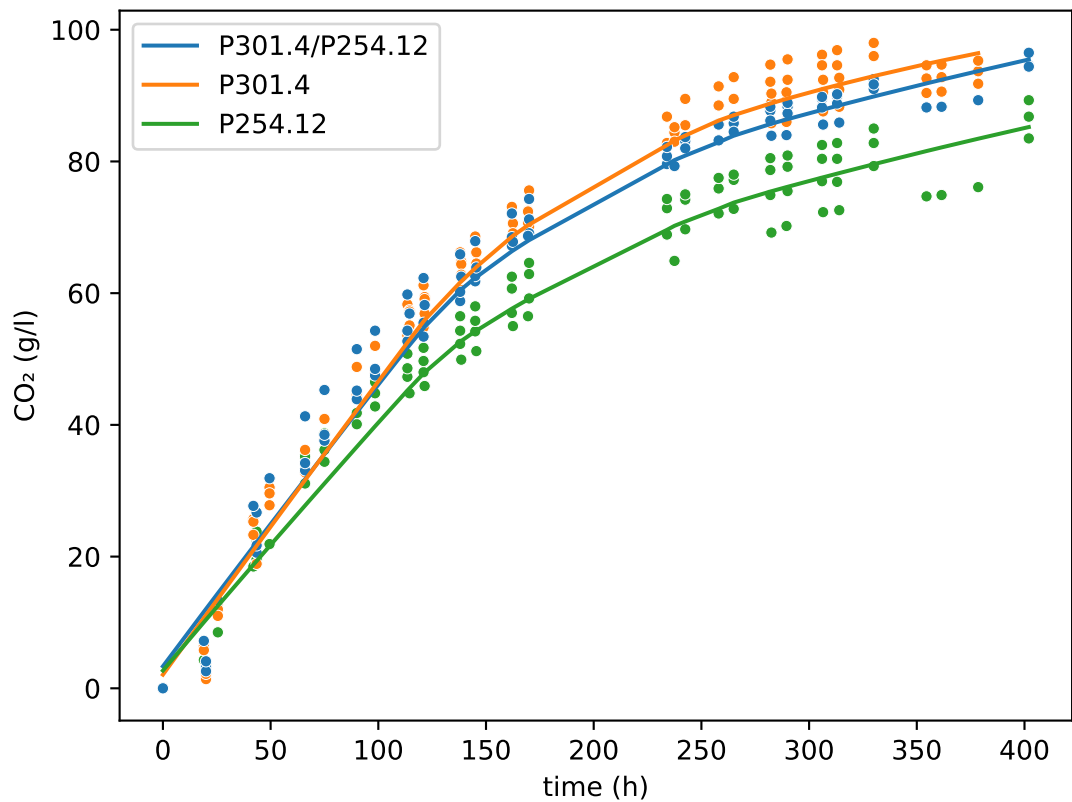

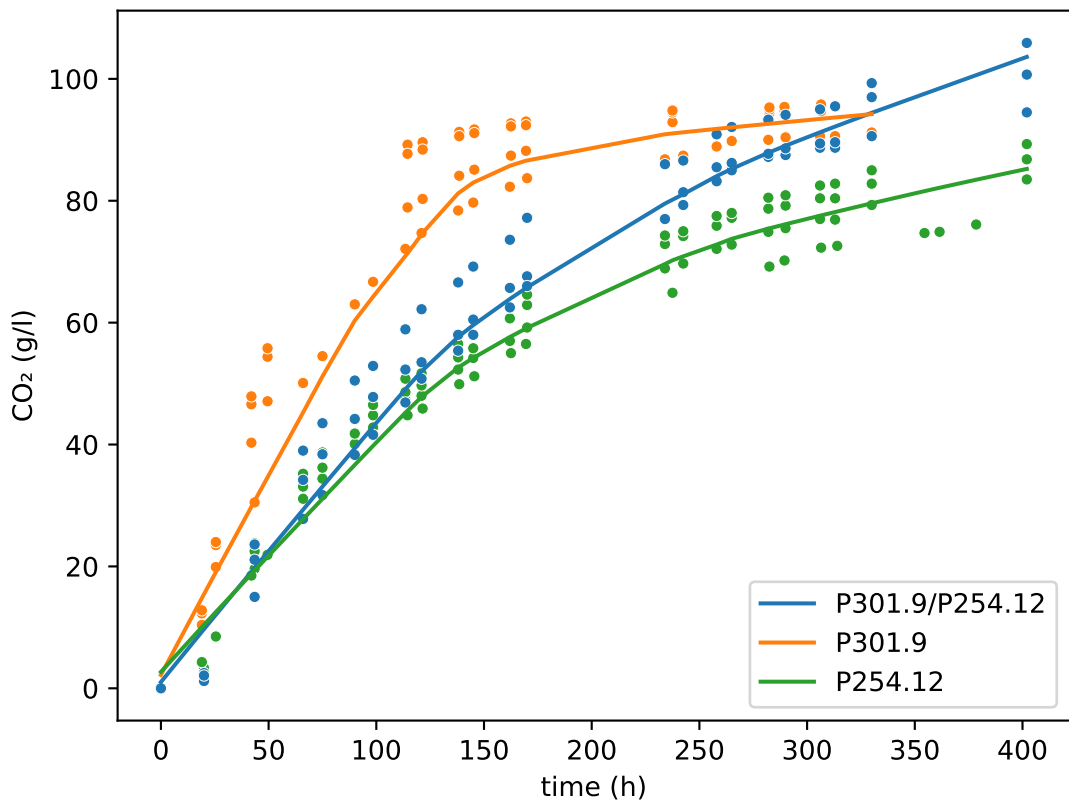

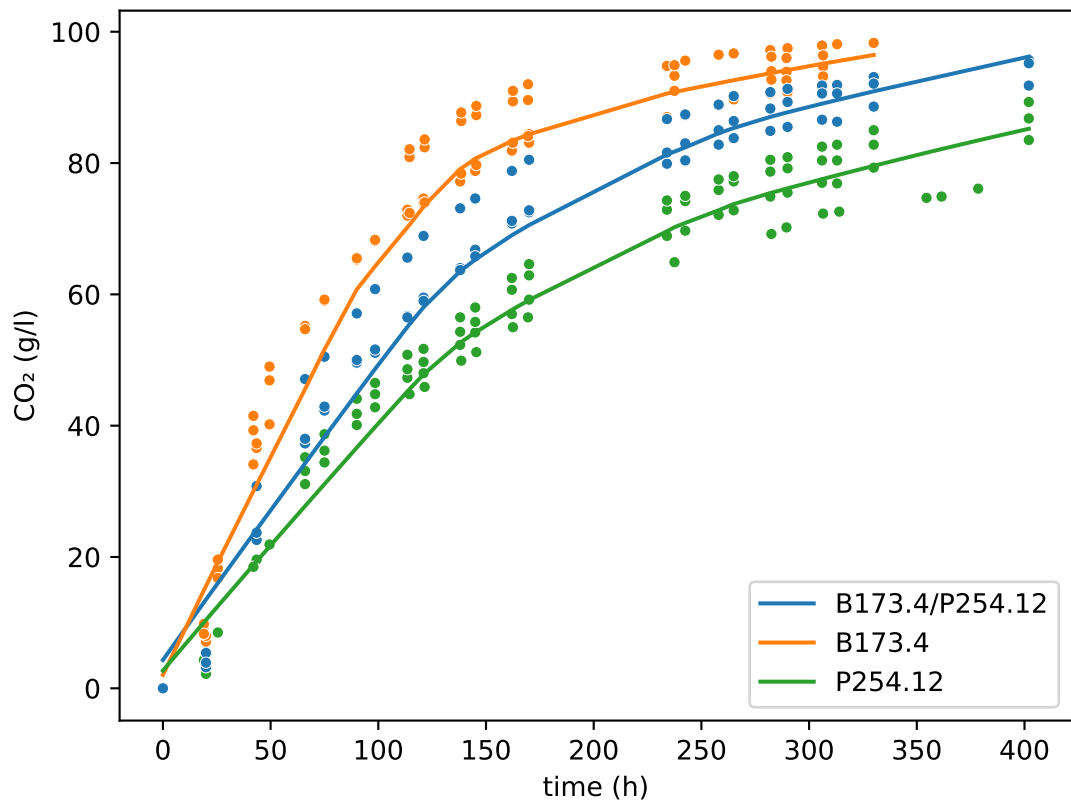

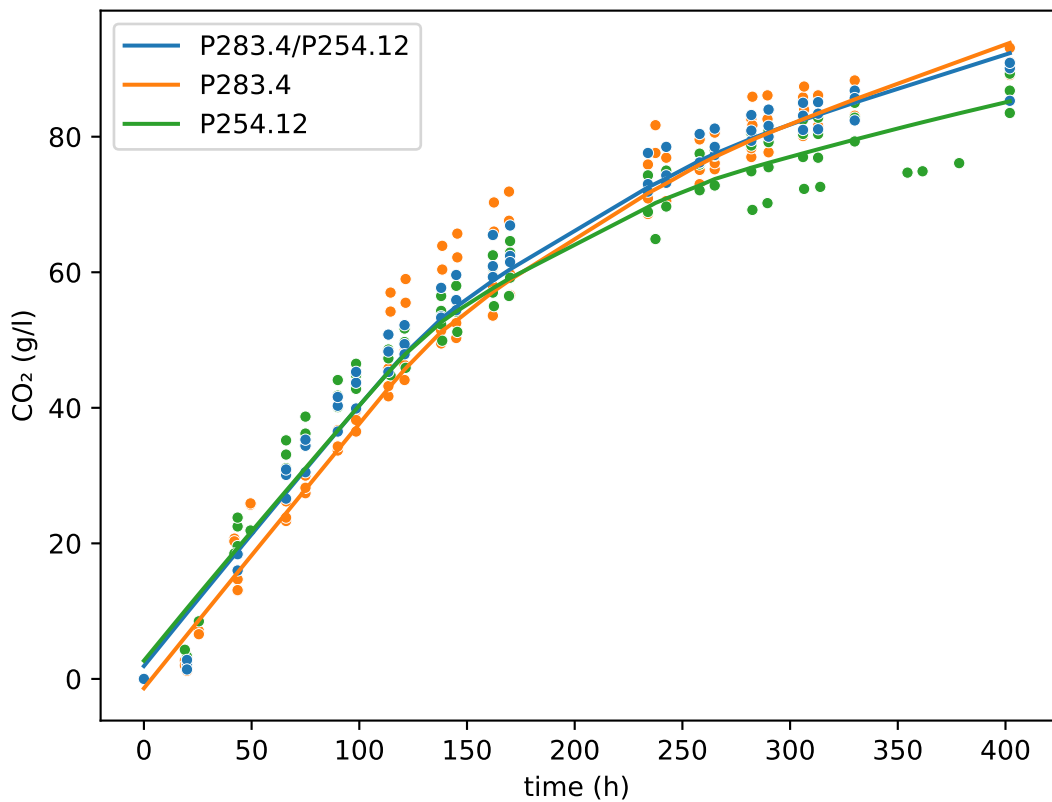

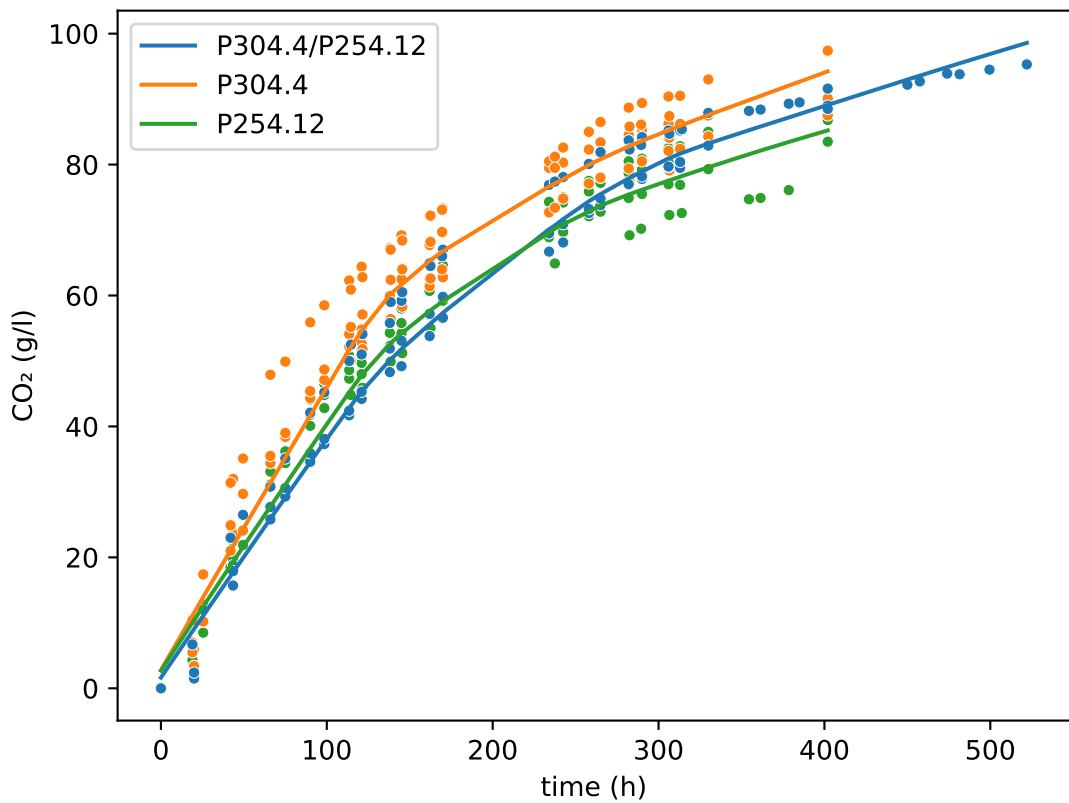

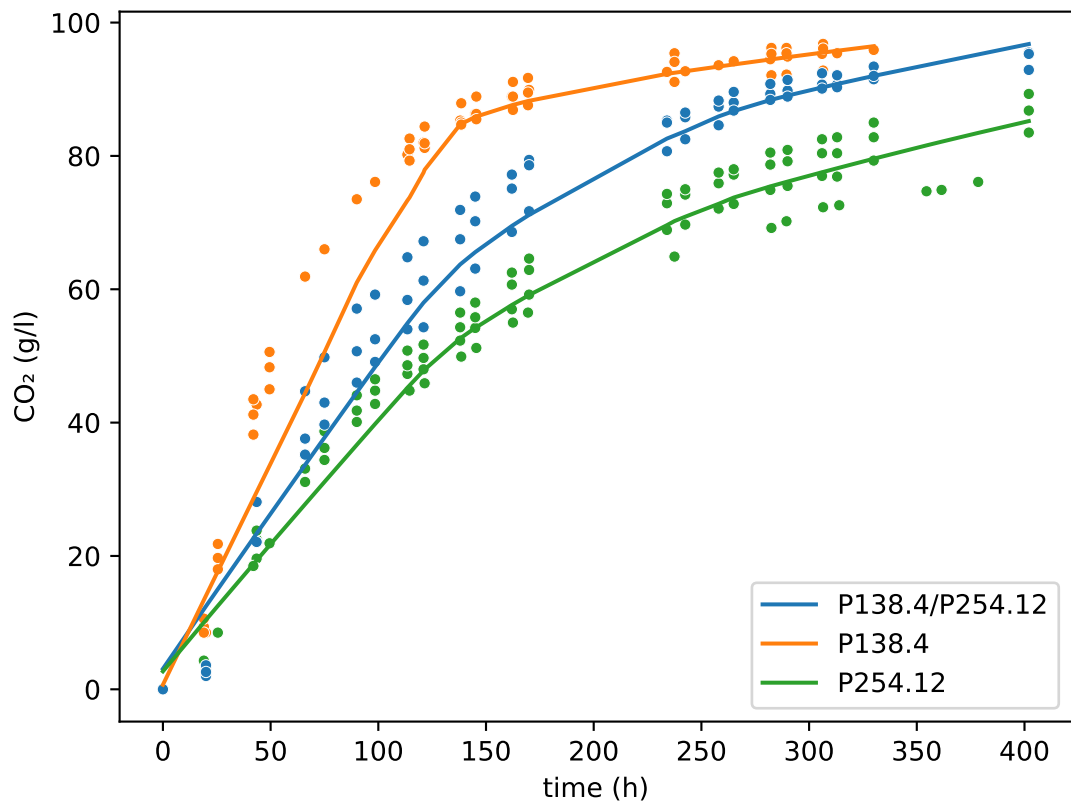

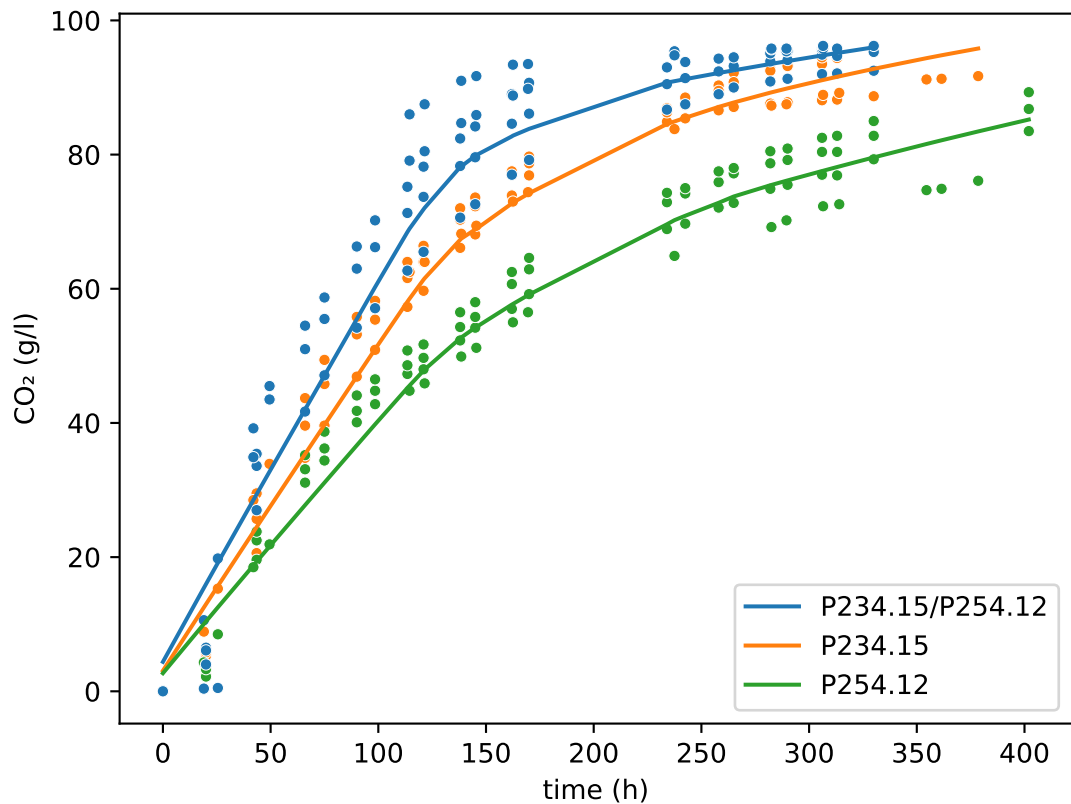

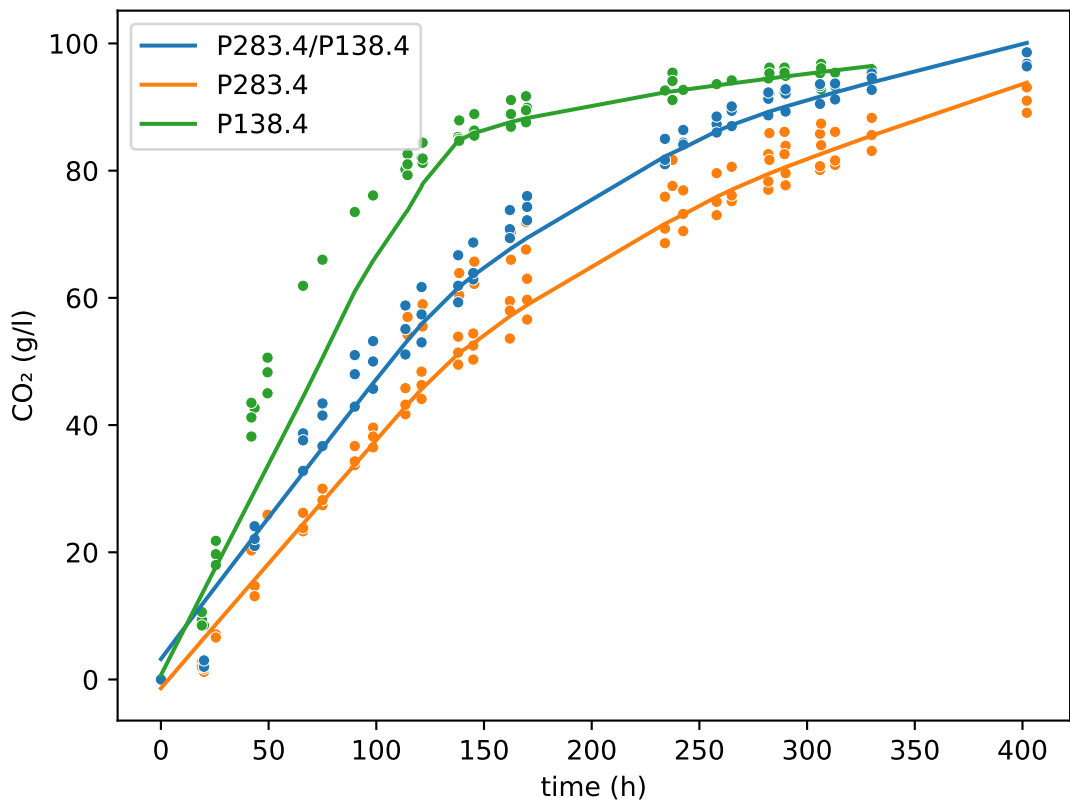

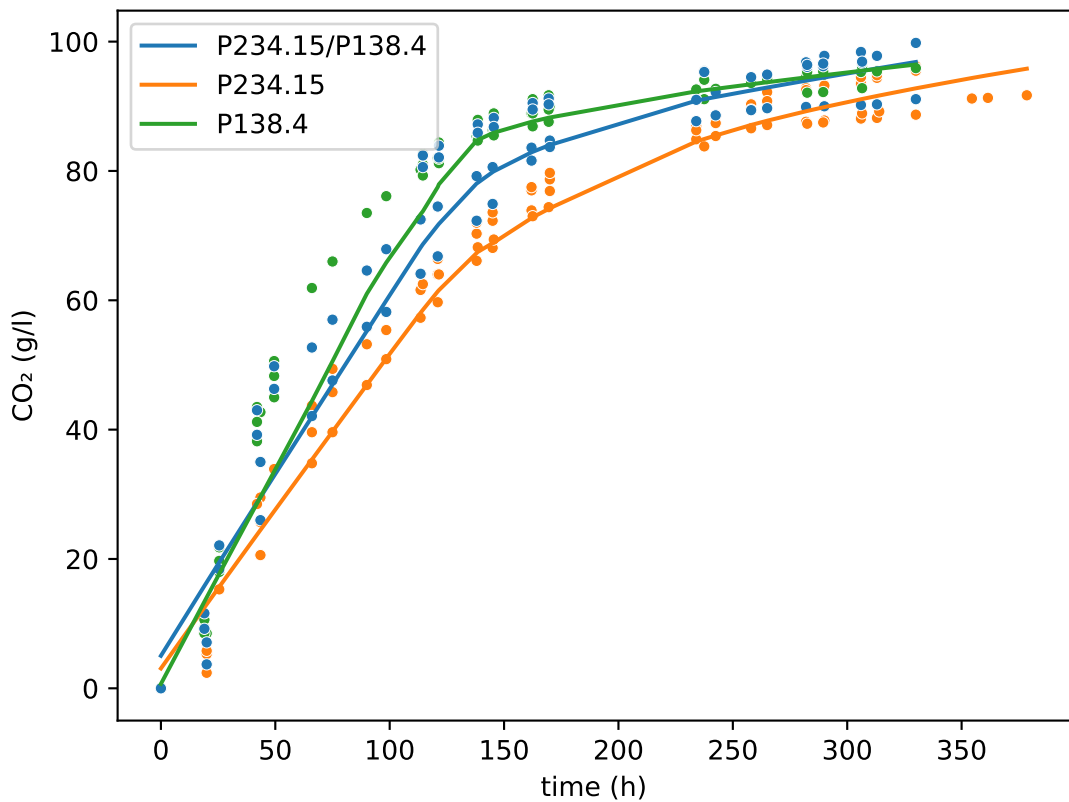

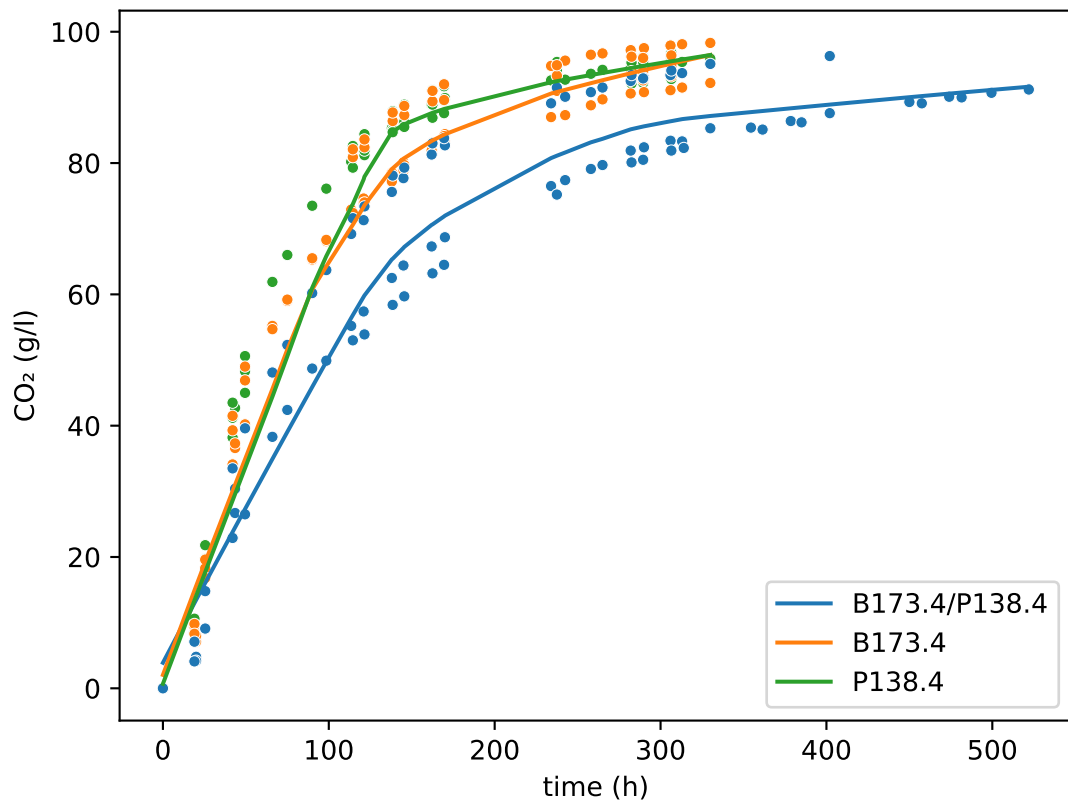

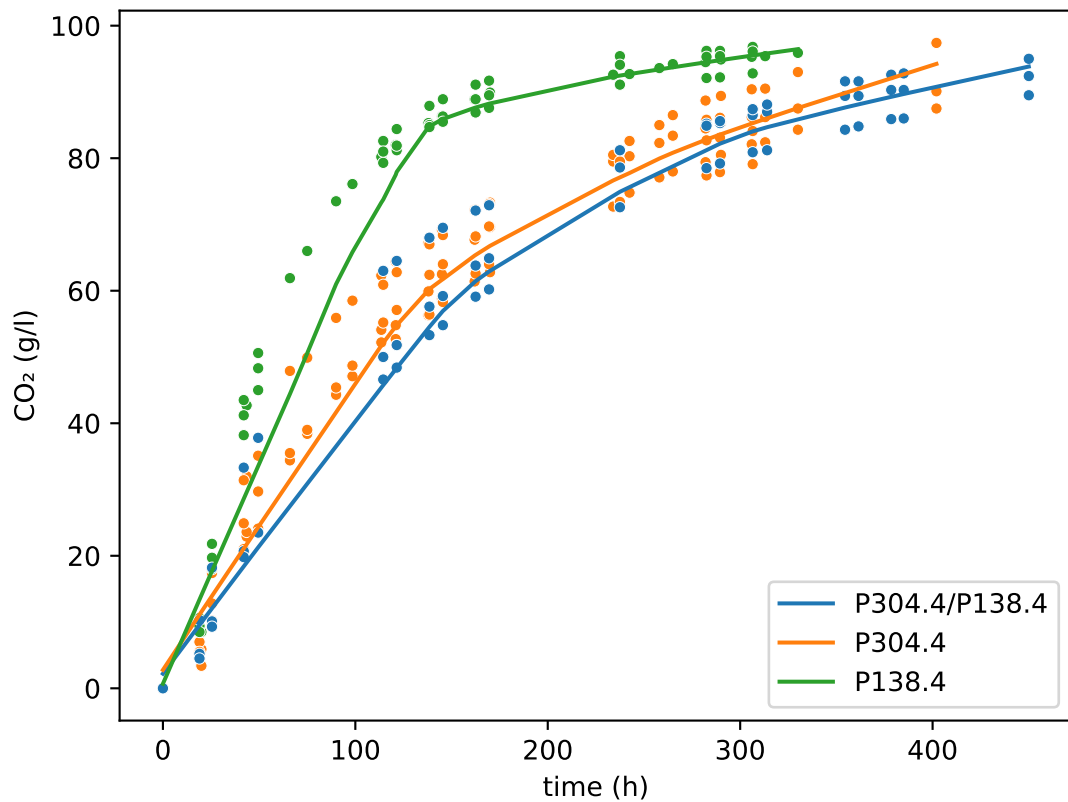

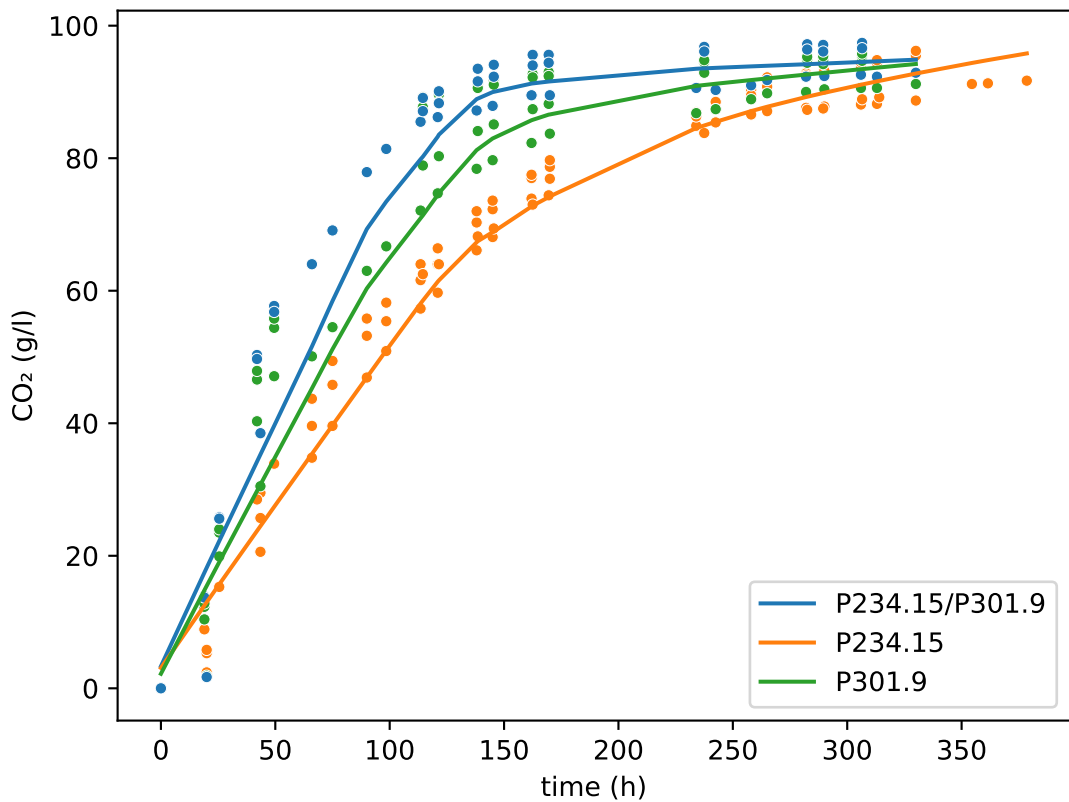

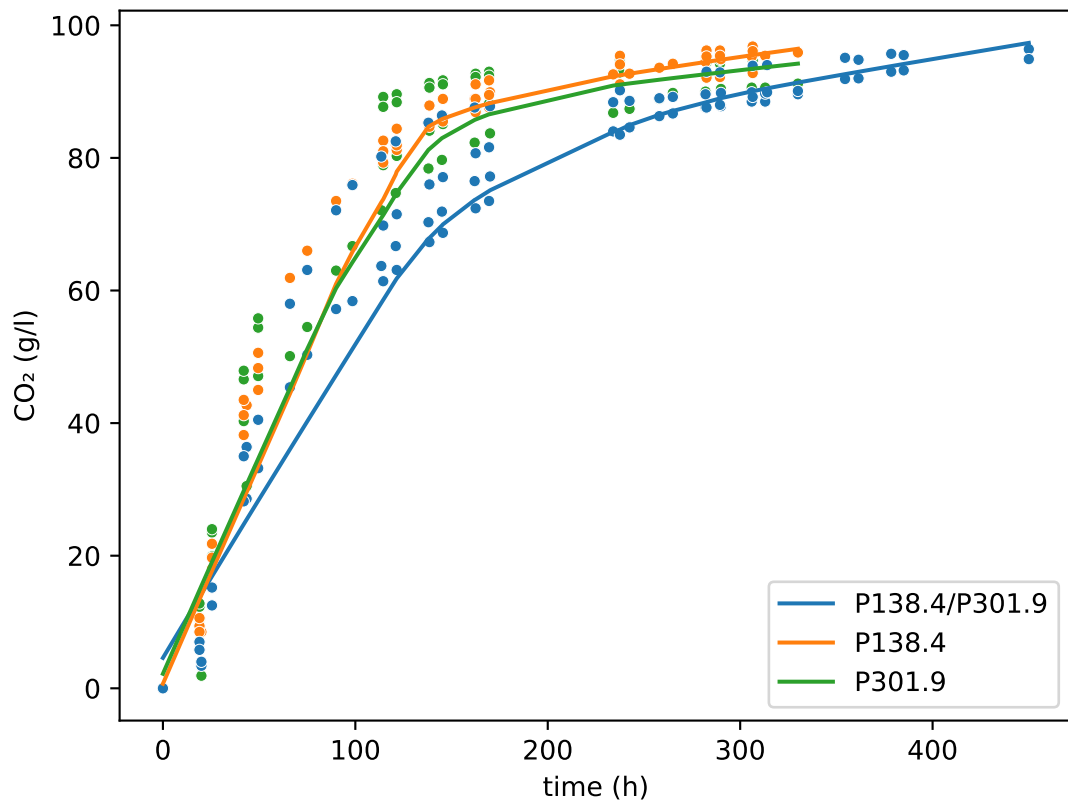

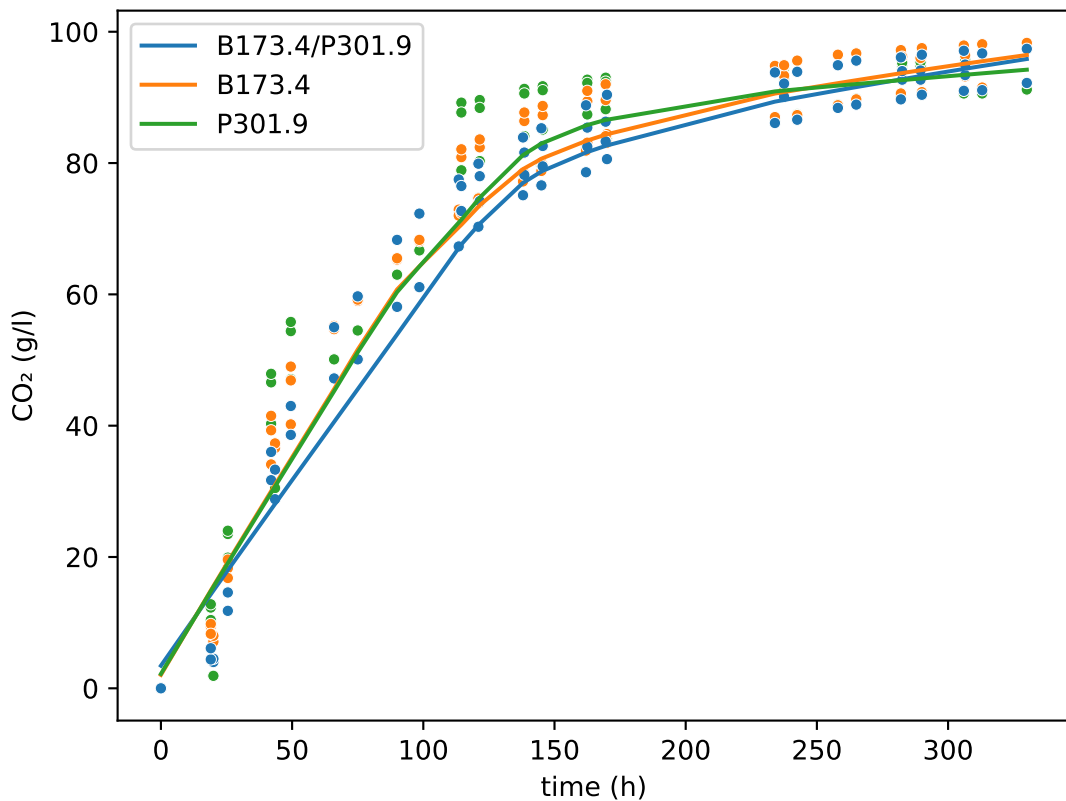

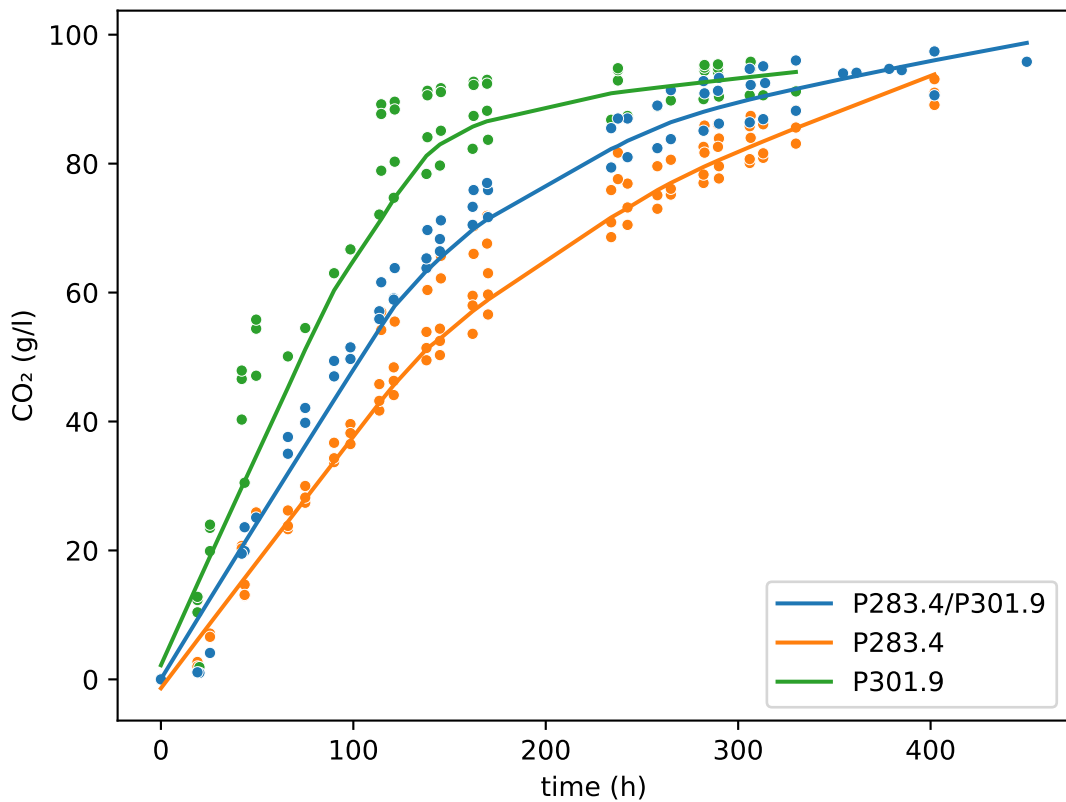

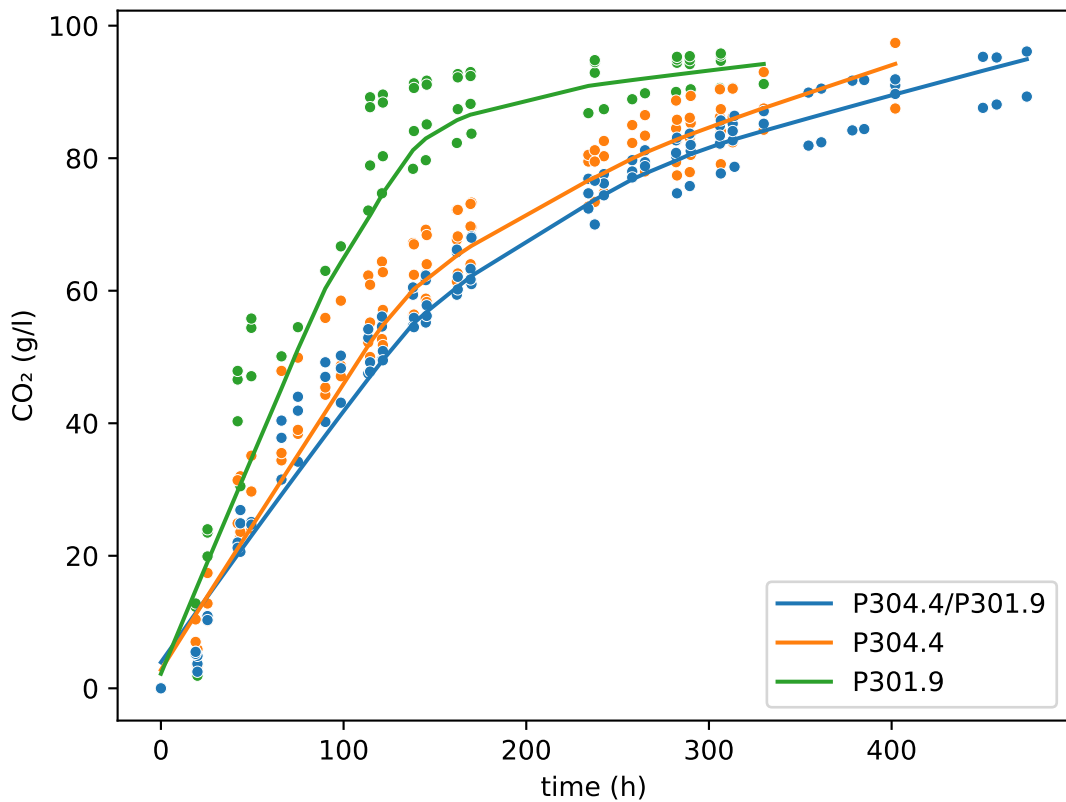

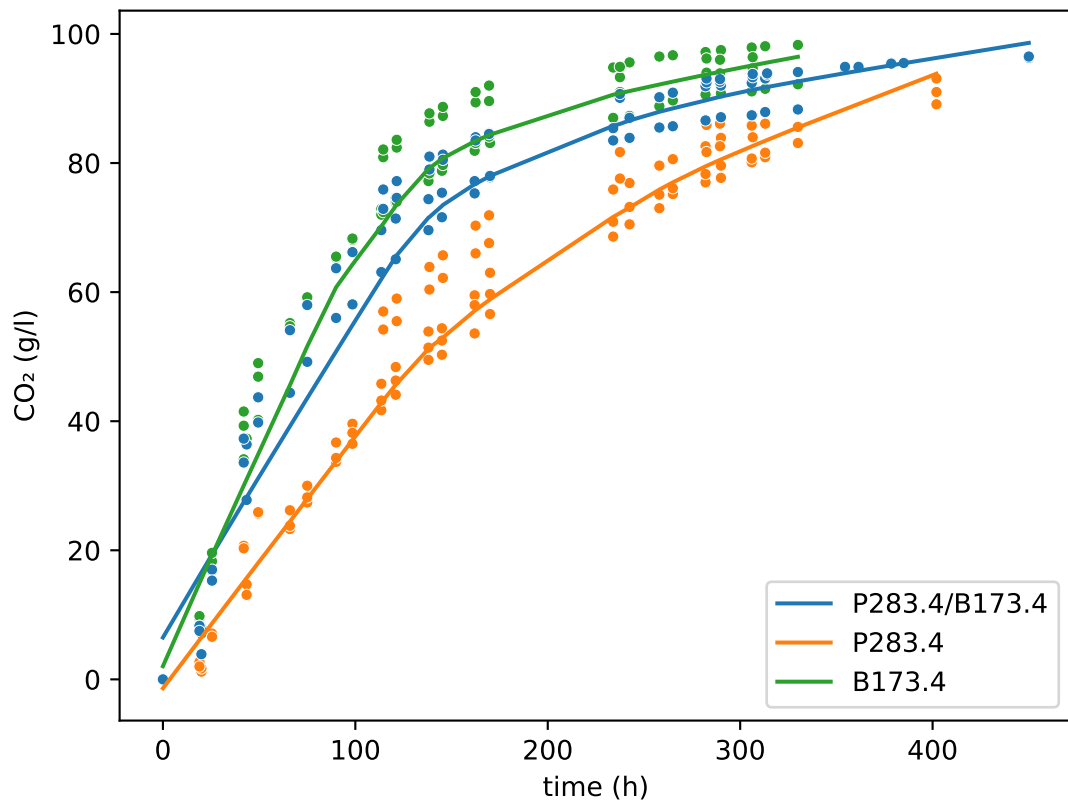

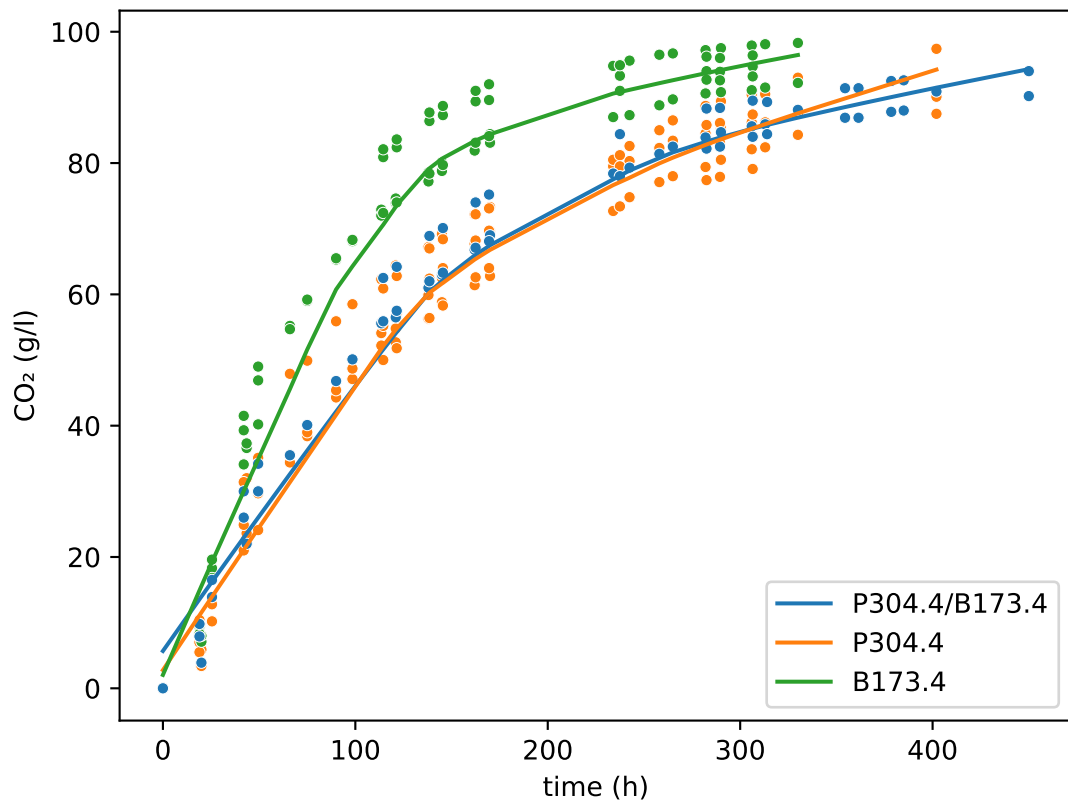

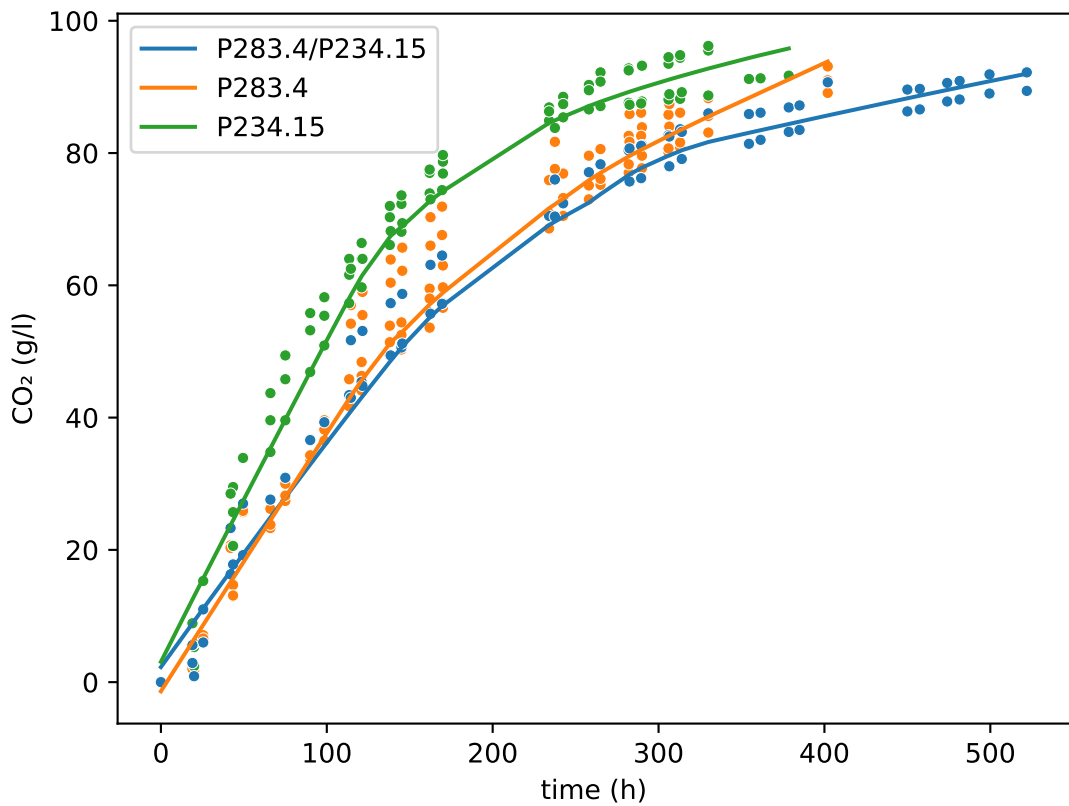

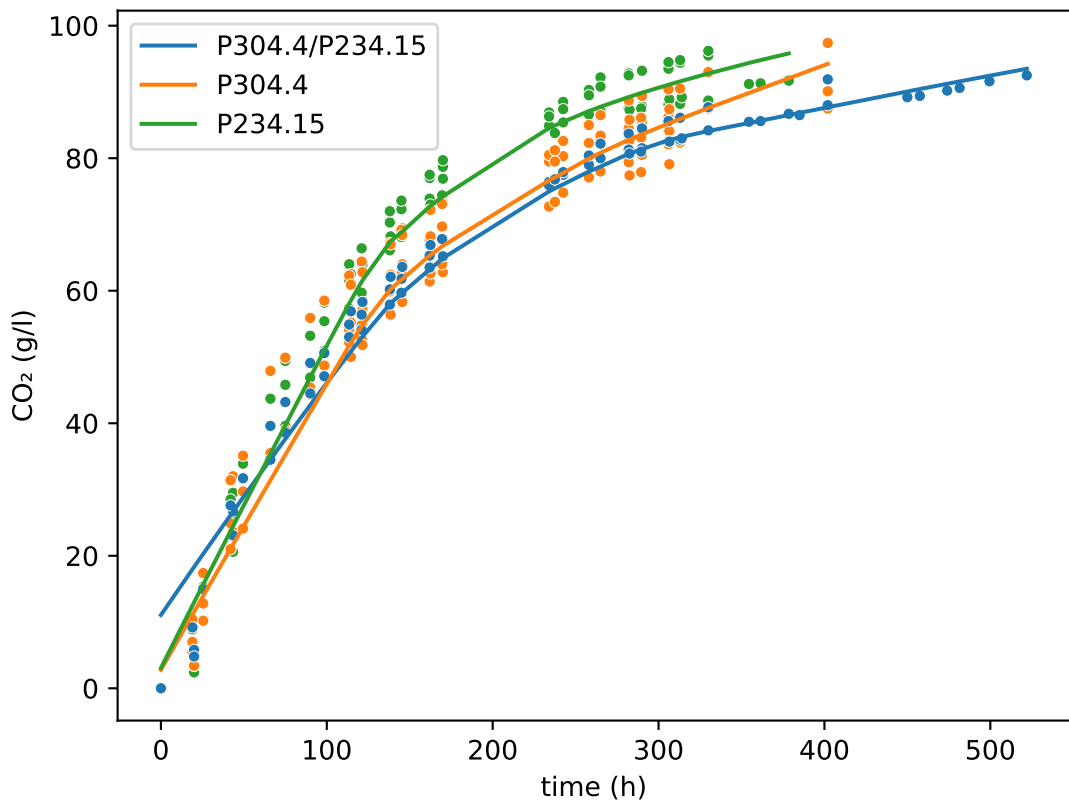

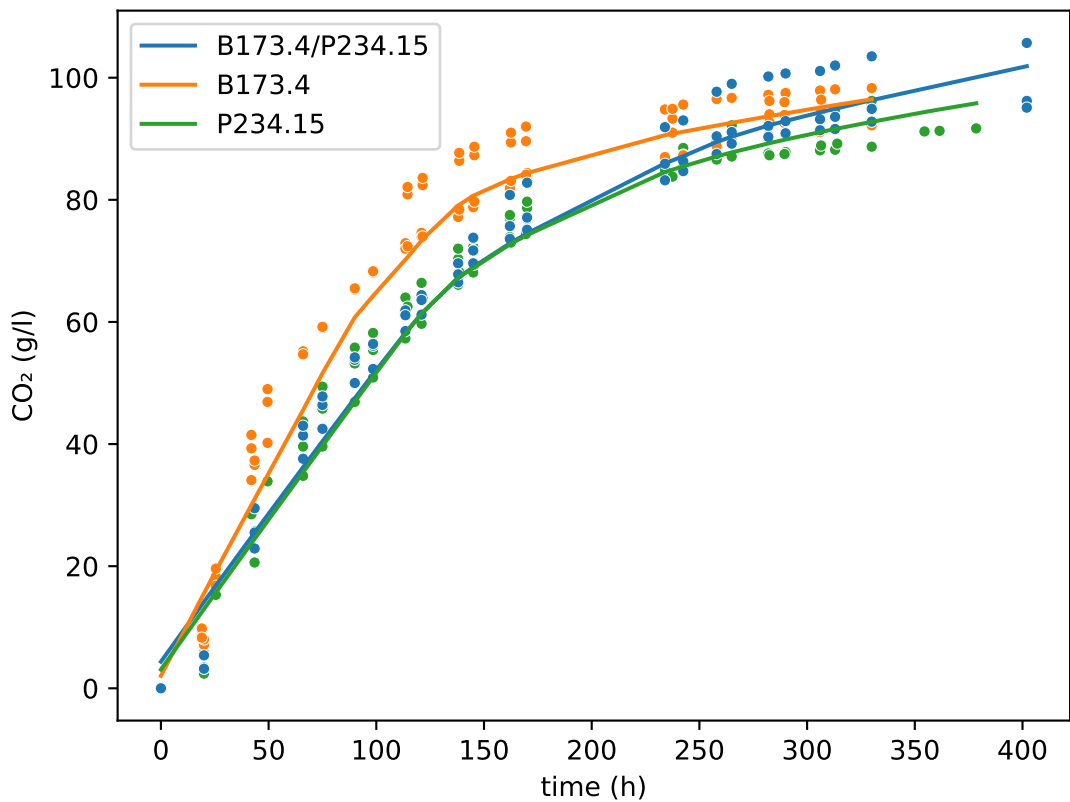

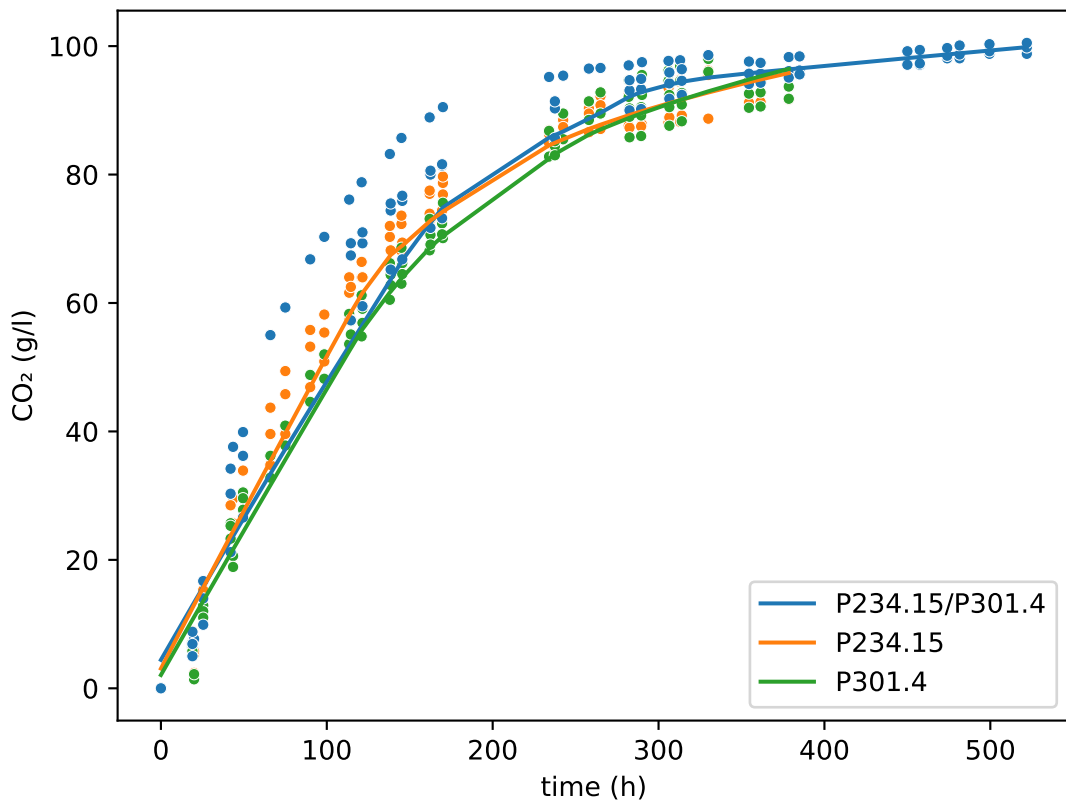

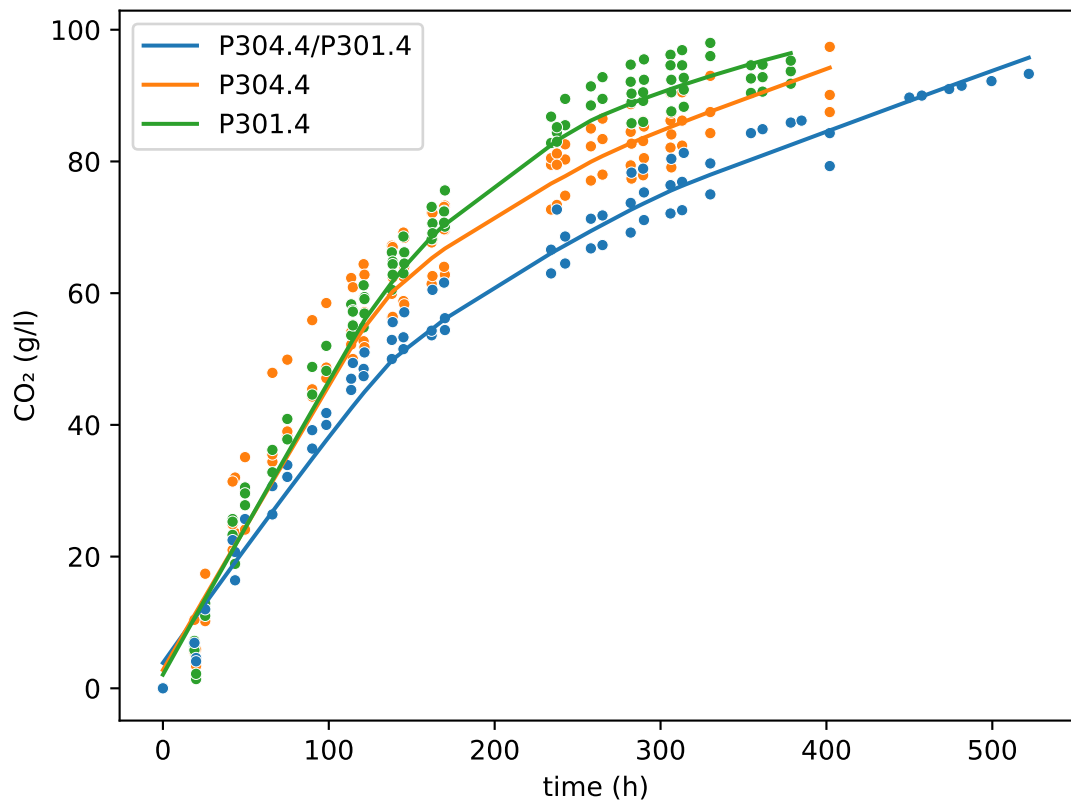

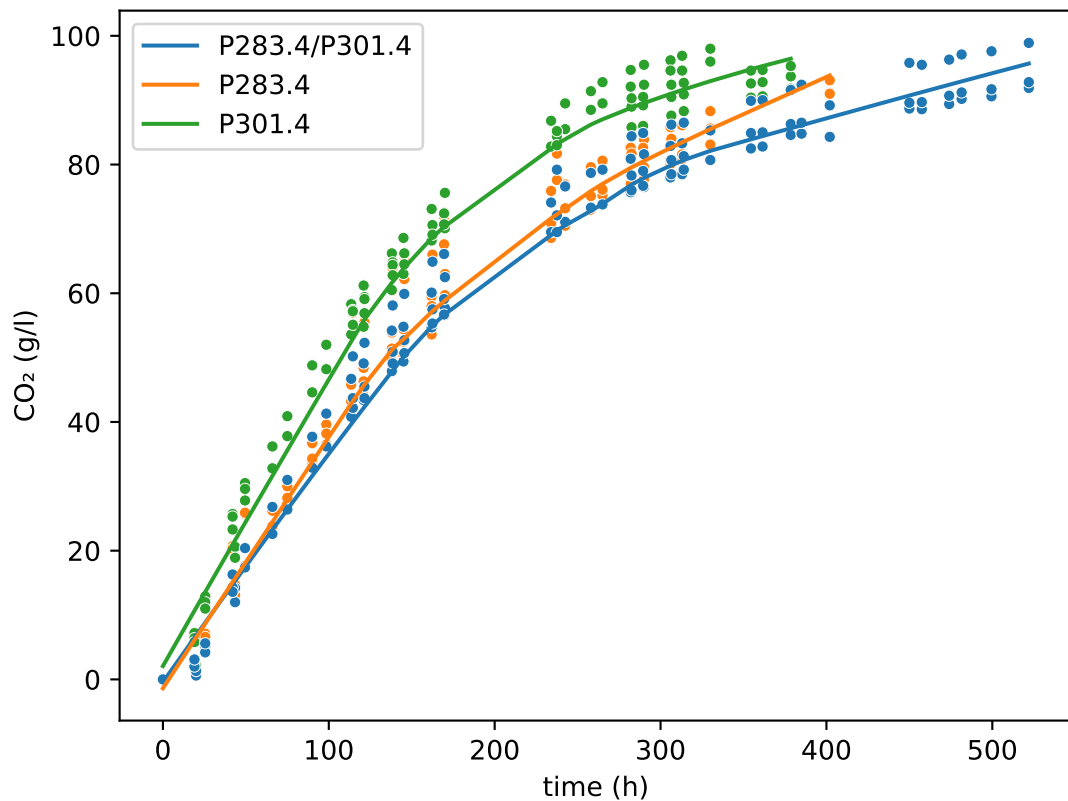

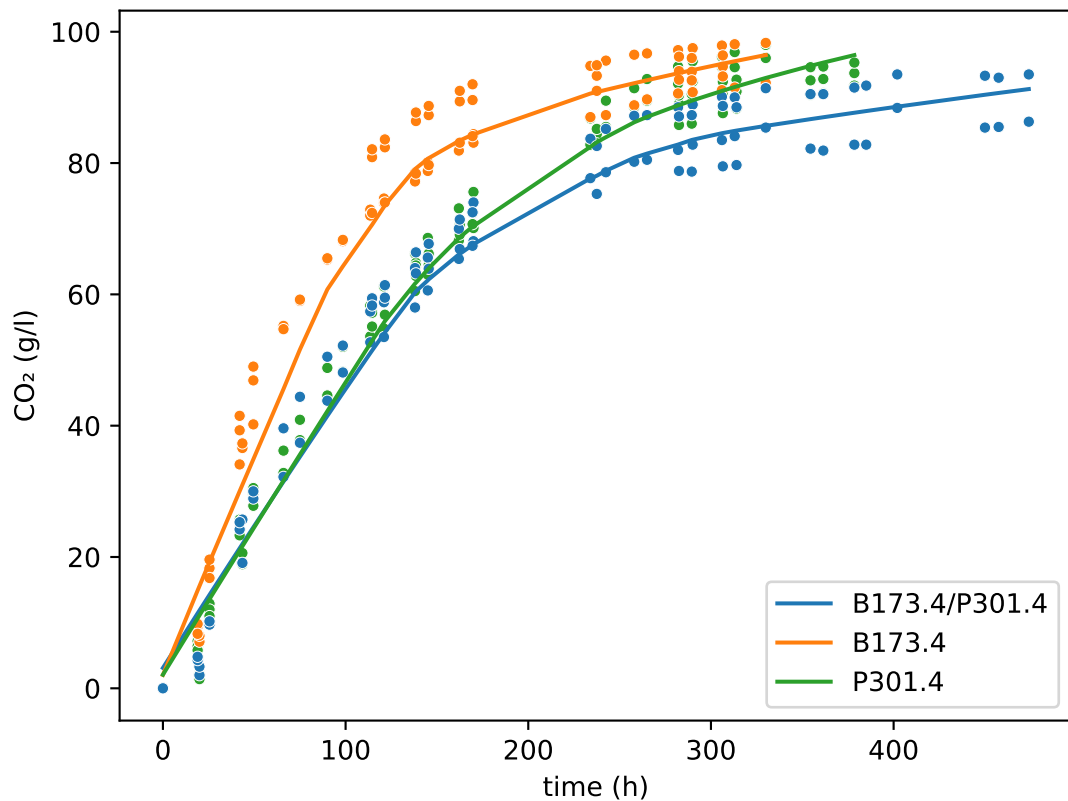

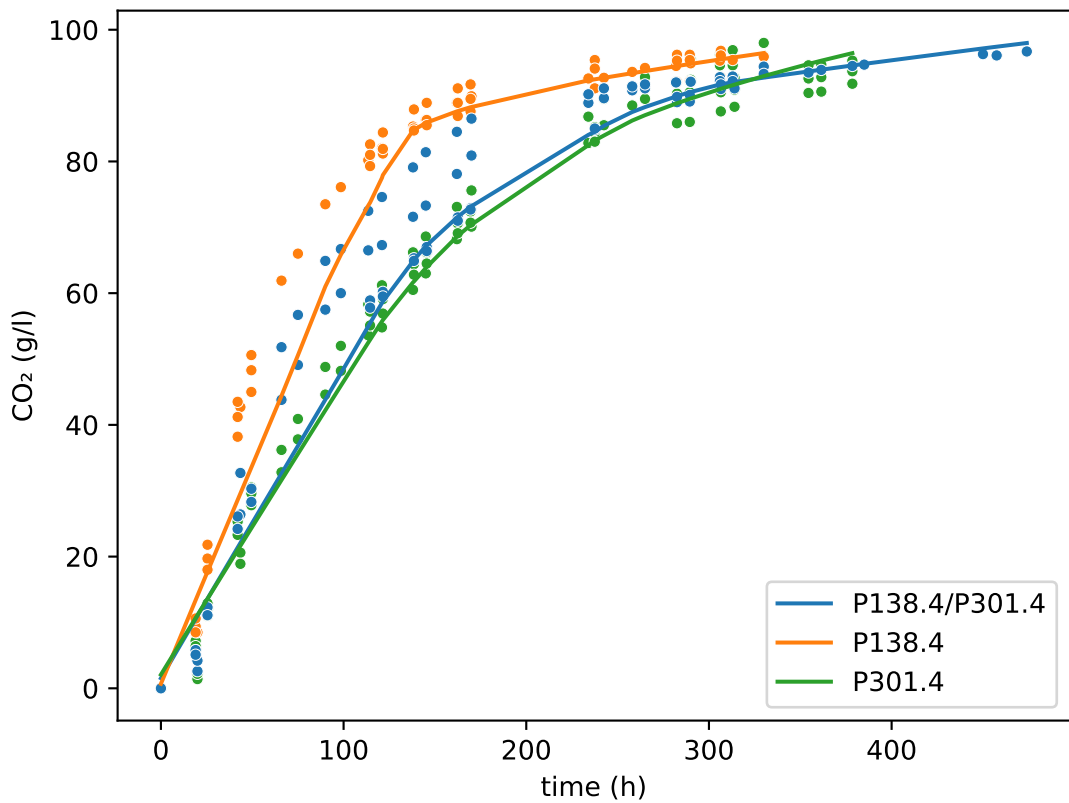

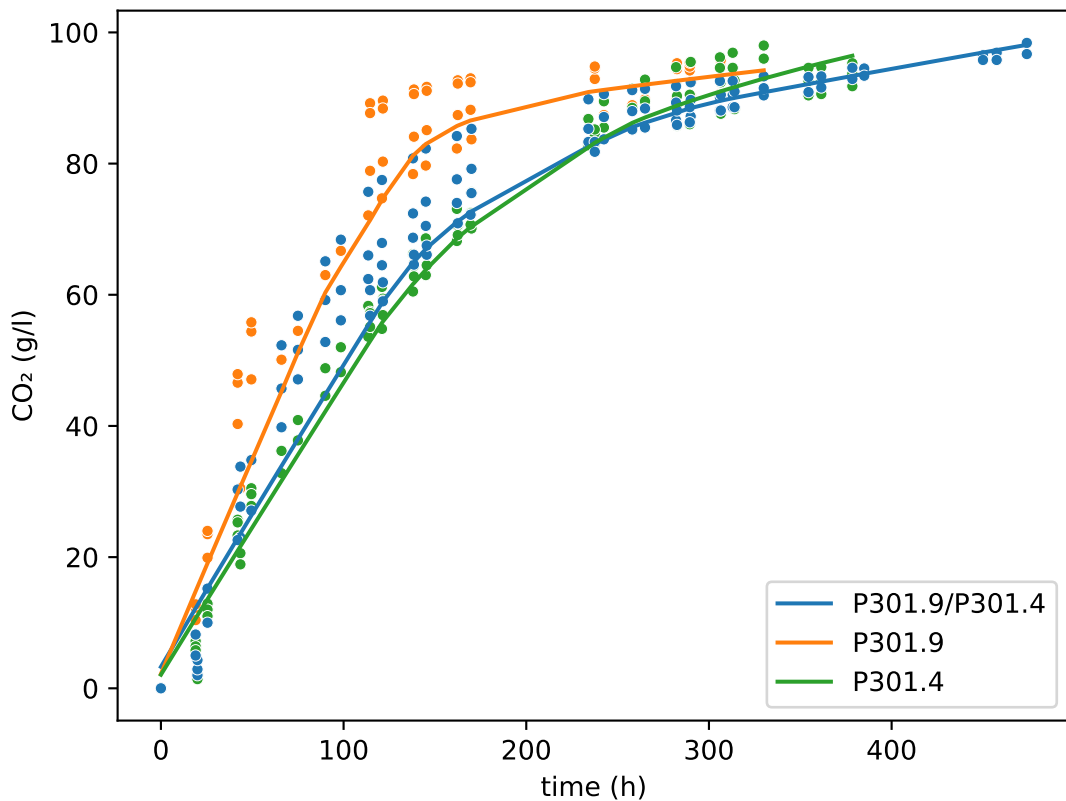

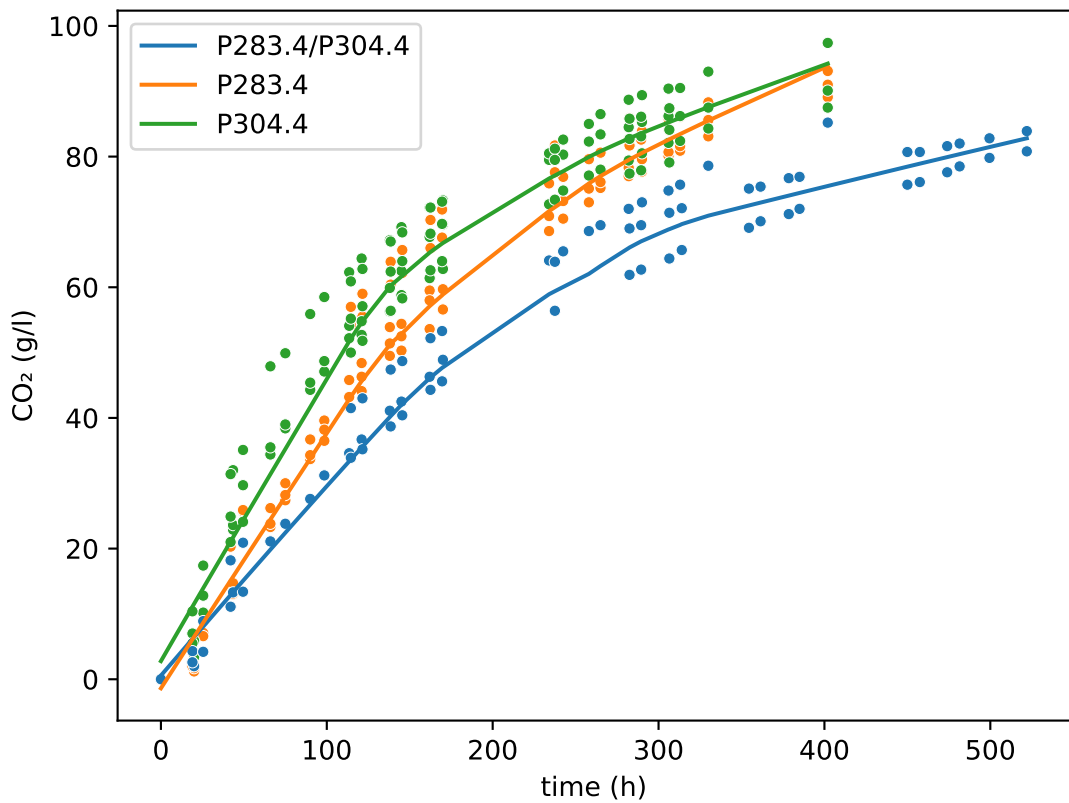

Supplement: S3 Fig — Each graph shows the kinetics trend of two single-strain fermentation kinetics (indicated by the strain name) and their respective co-fermentation kinetics (indicated by the strain names separated by a slash). (PDF) [file pone.0300212.s003.pdf]
