# Supplementary material for: Strain prevalence and killer factor only partially influence the fermentation activity of pairwise Saccharomyces cerevisiae wine strains inoculation
Source: PLoS One. 2024 Apr 29;19(4):e0300212. doi: 10.1371/journal.pone.0300212 (PMC11057759; doi:10.1371/journal.pone.0300212)
Supplement: S2 Fig — The name of the strain tested for sensitivity (i.e the strain inoculated in the whole medium) is indicated in the upper left corner of each picture. (PDF) [file pone.0300212.s002.pdf]

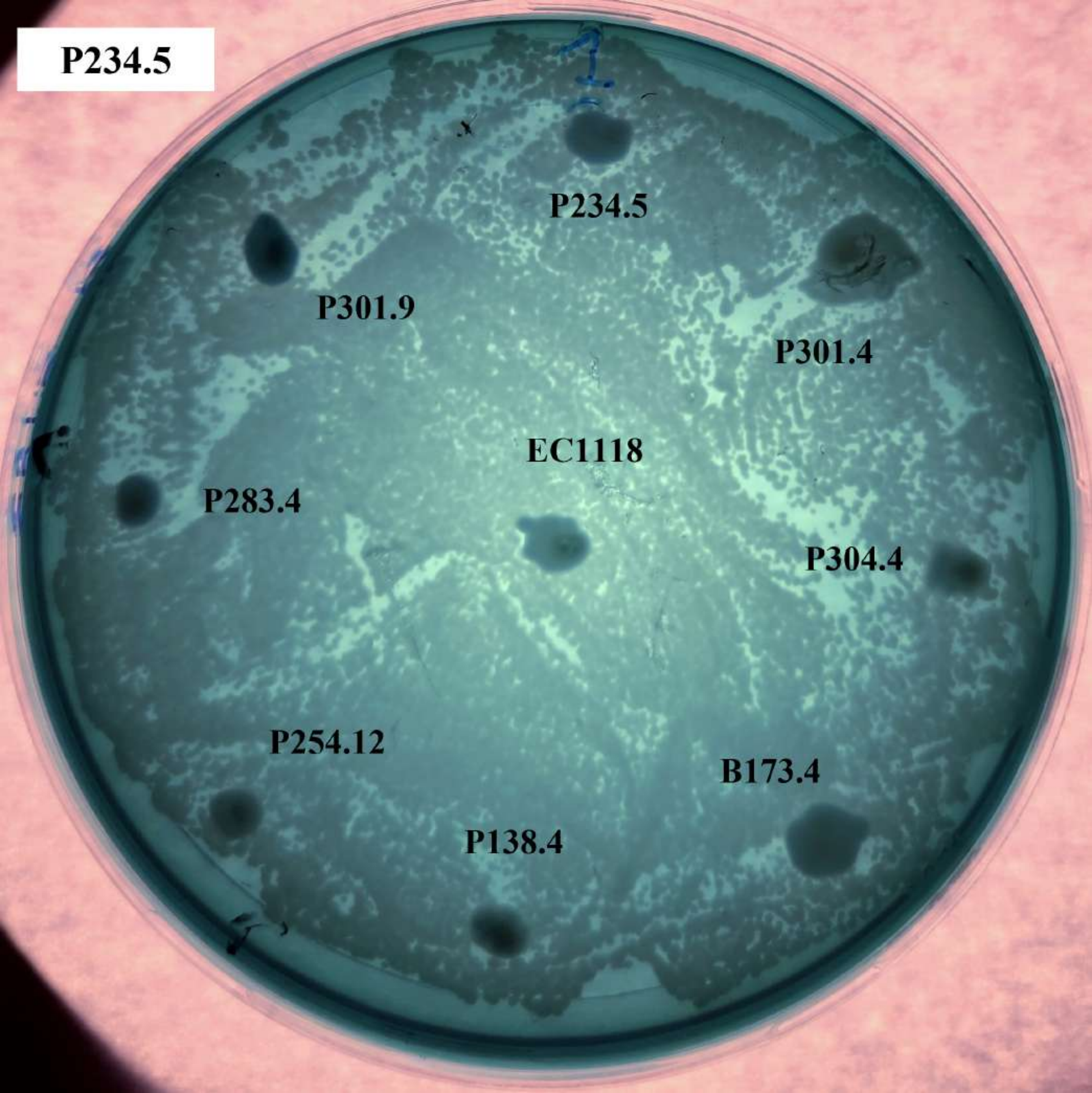
P234.5
P234.5
P301.9
P301.4
EC1118
P283.4
P304.4
P254.12
B173.4
P138.4

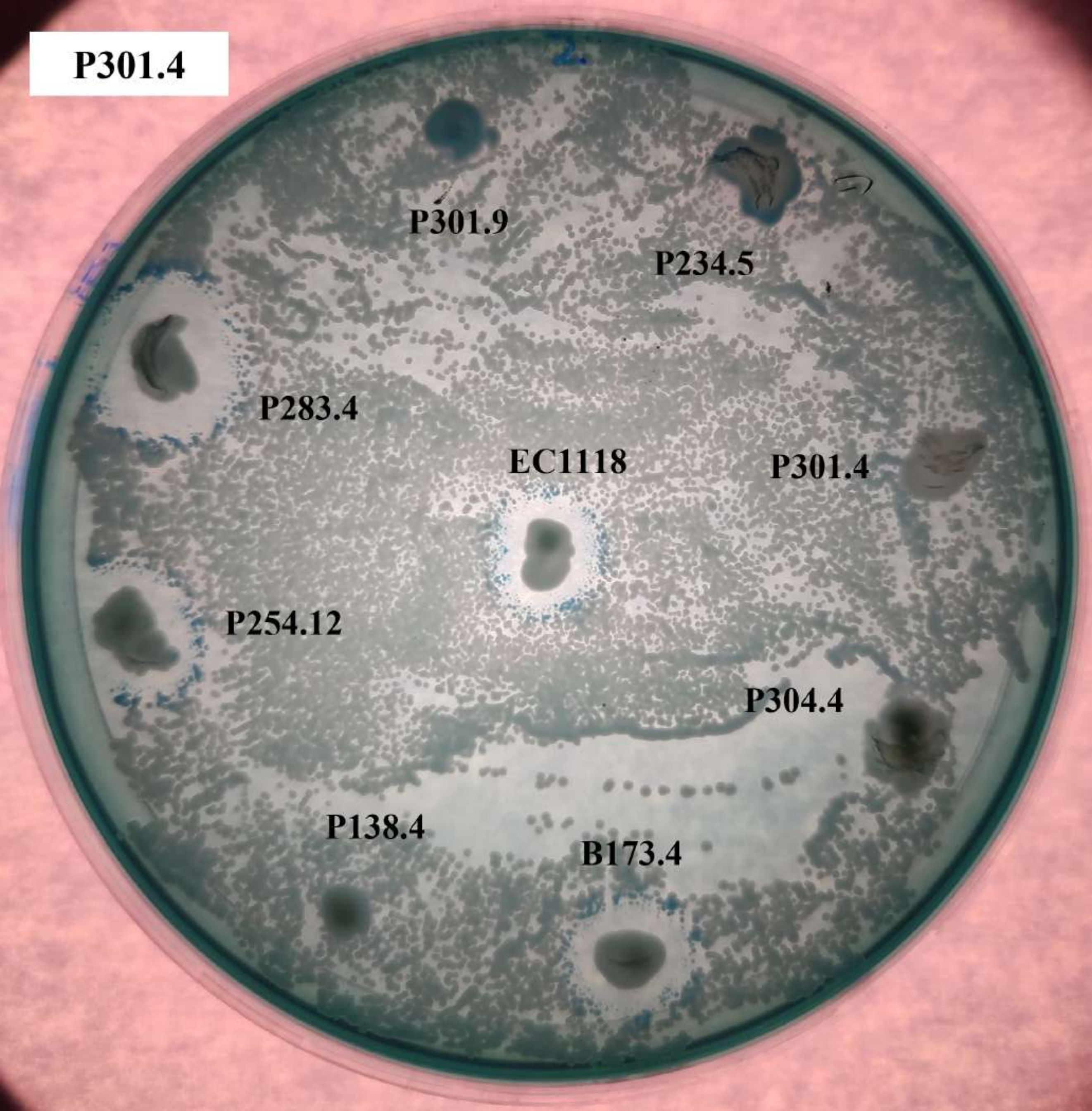
P301.4
P301.9
P234.5
P283.4
EC1118
P301.4
P254.12
P304.4
P138.4
B173.4

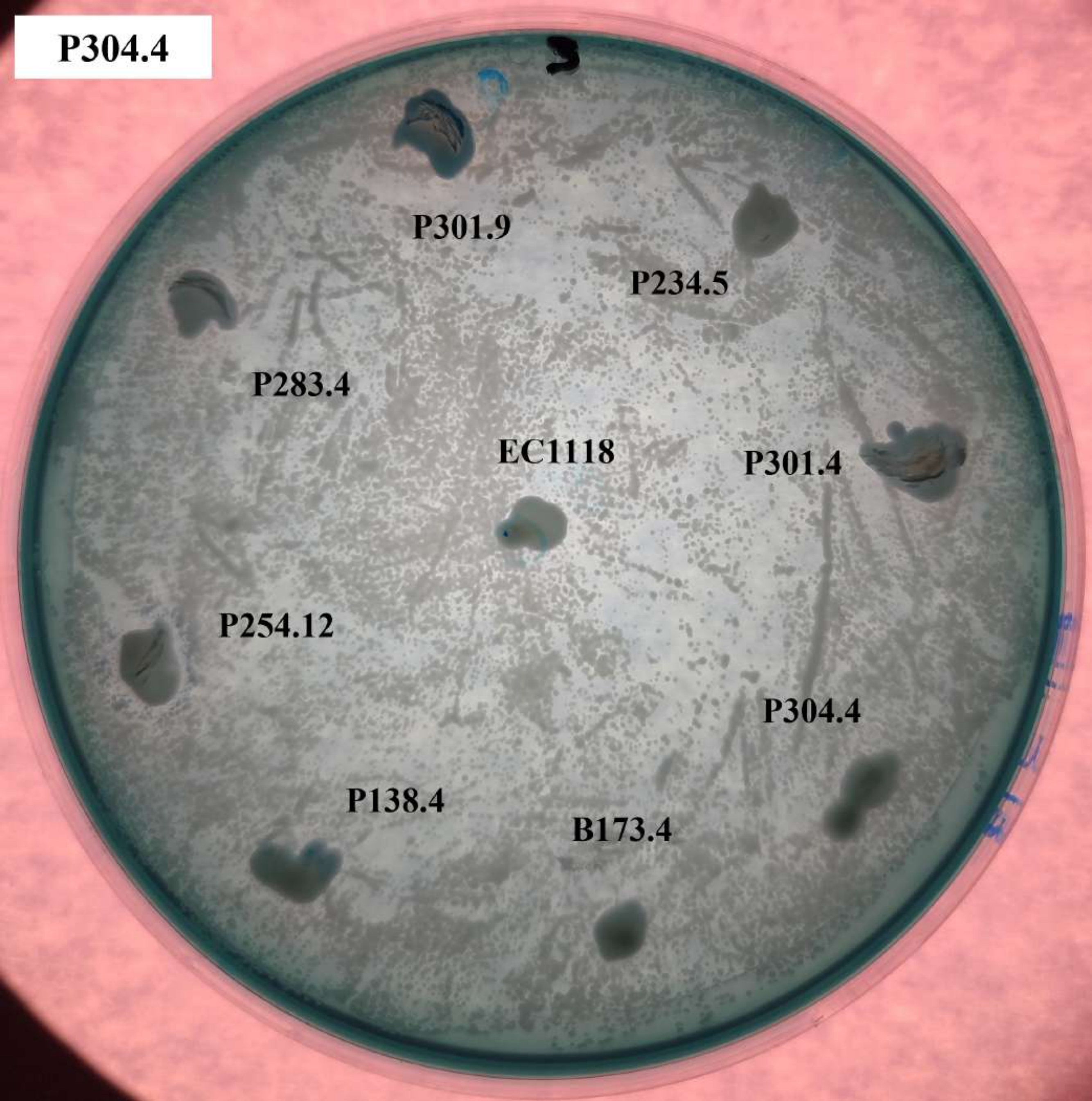
P304.4
P301.9
P234.5
P283.4
EC1118
P301.4
P254.12
P304.4
P138.4
B173.4

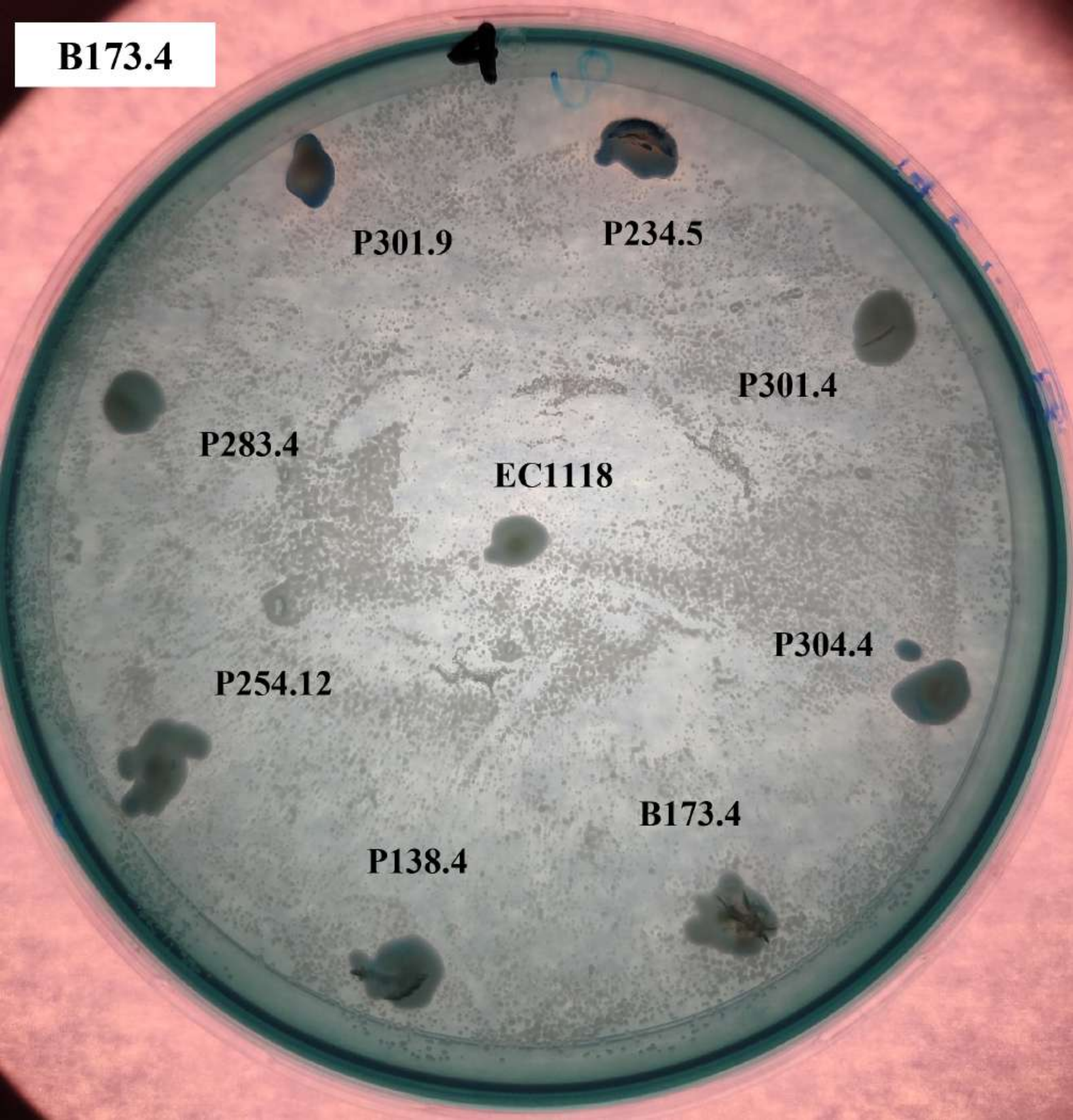

B173.4
P301.9
P234.5
P301.4
P283.4
EC1118
P304.4
P254.12
B173.4
P138.4

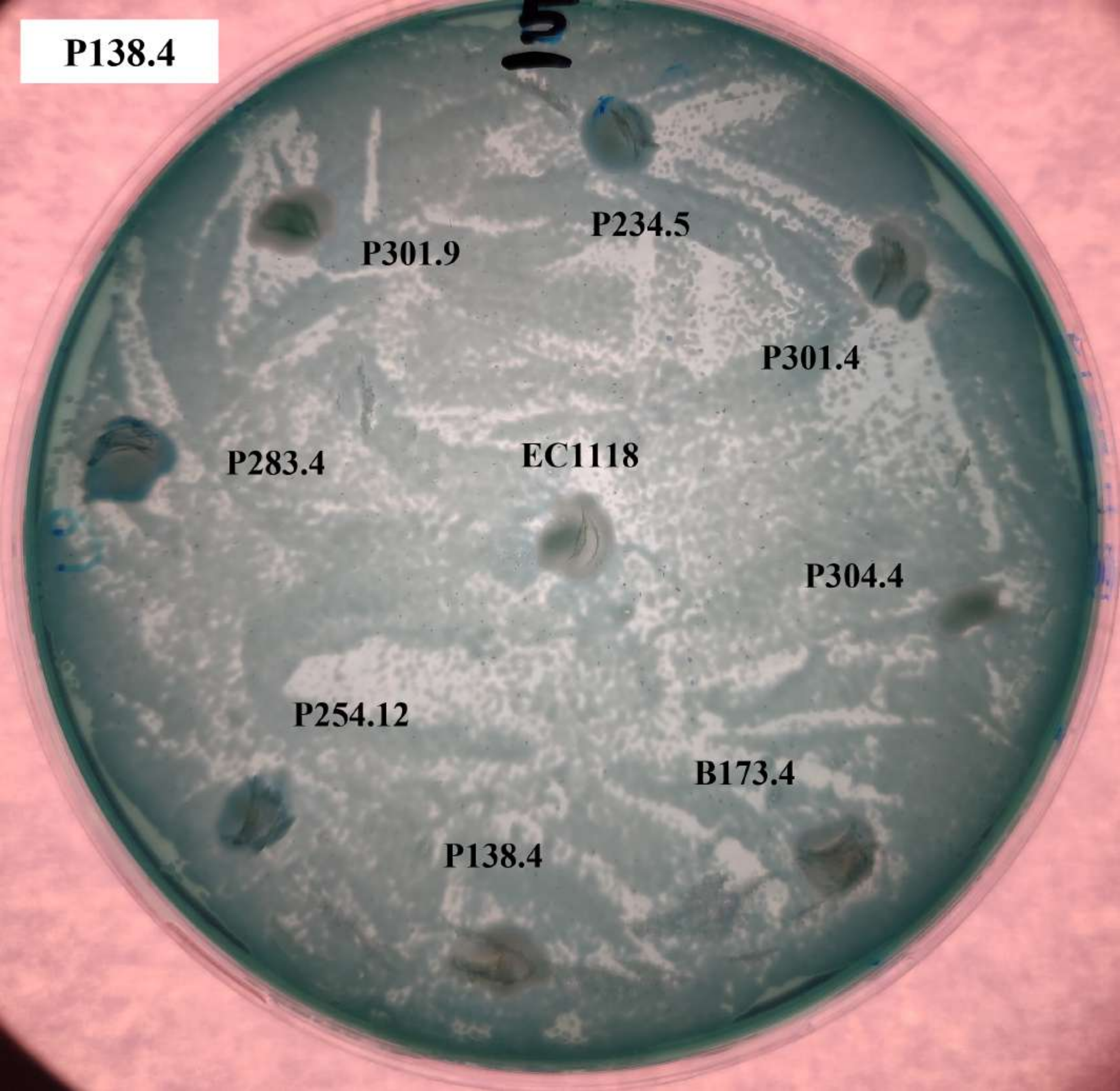
P138.4
P234.5
P301.9
P301.4
P283.4
EC1118
P304.4
P254.12
B173.4
P138.4

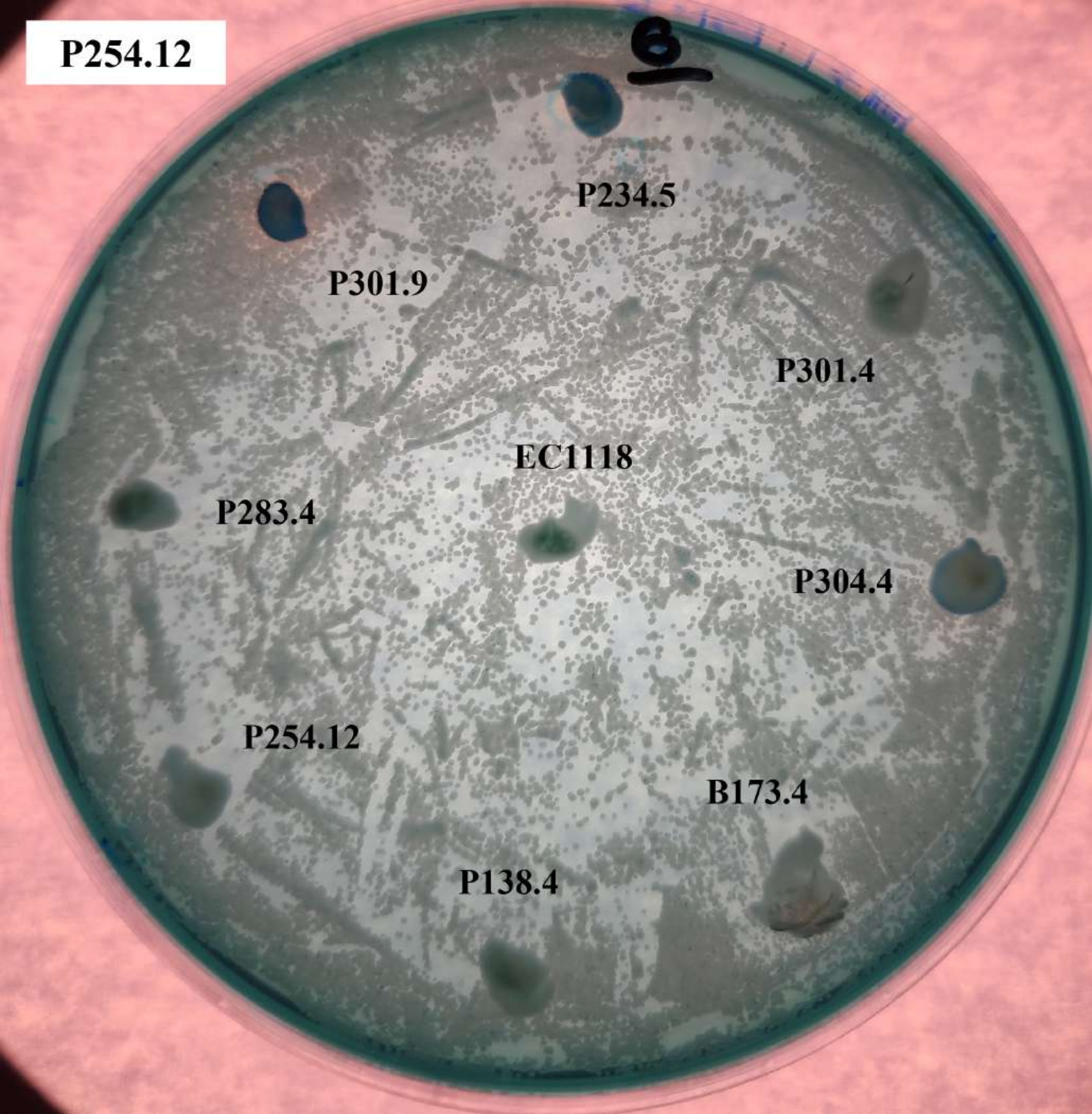

P254.12
6
P234.5
P301.9
P301.4
EC1118
P283.4
P304.4
P254.12
B173.4
P138.4

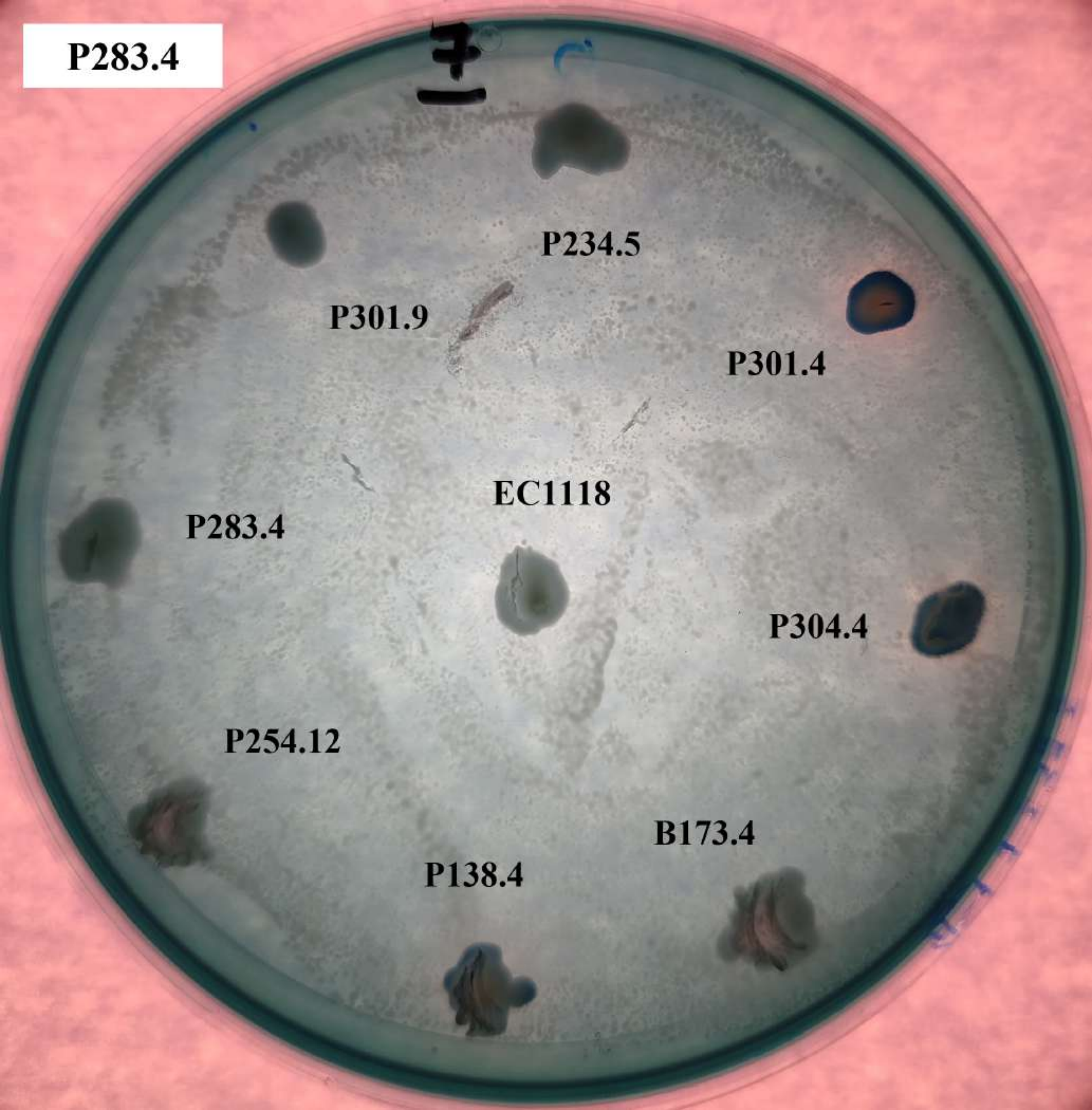
P283.4
P234.5
P301.9
P301.4
EC1118
P283.4
P304.4
P254.12
B173.4
P138.4

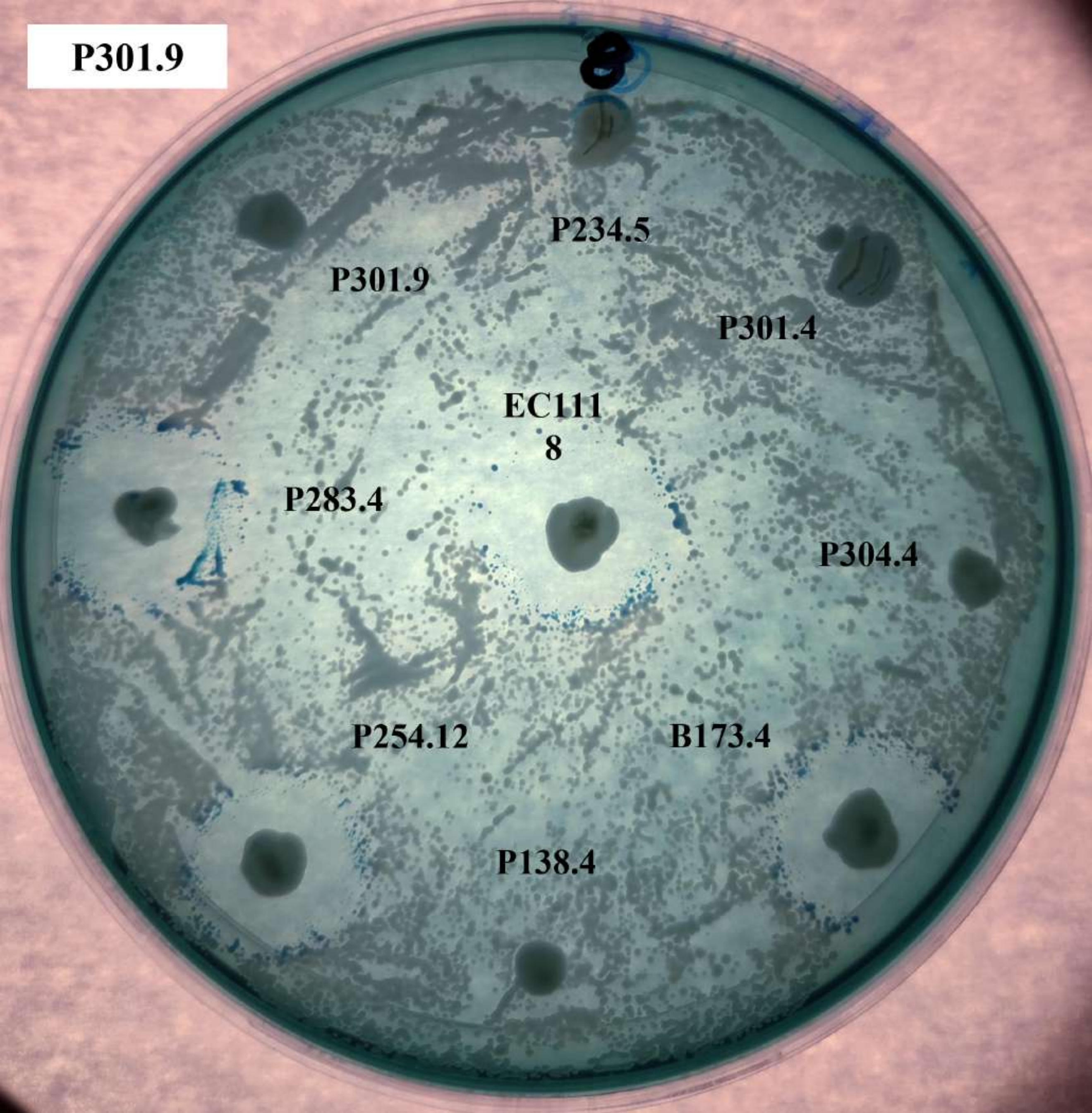
P301.9
P234.5
P301.9
P301.4
EC111
8
P283.4
P304.4
P254.12
B173.4
P138.4

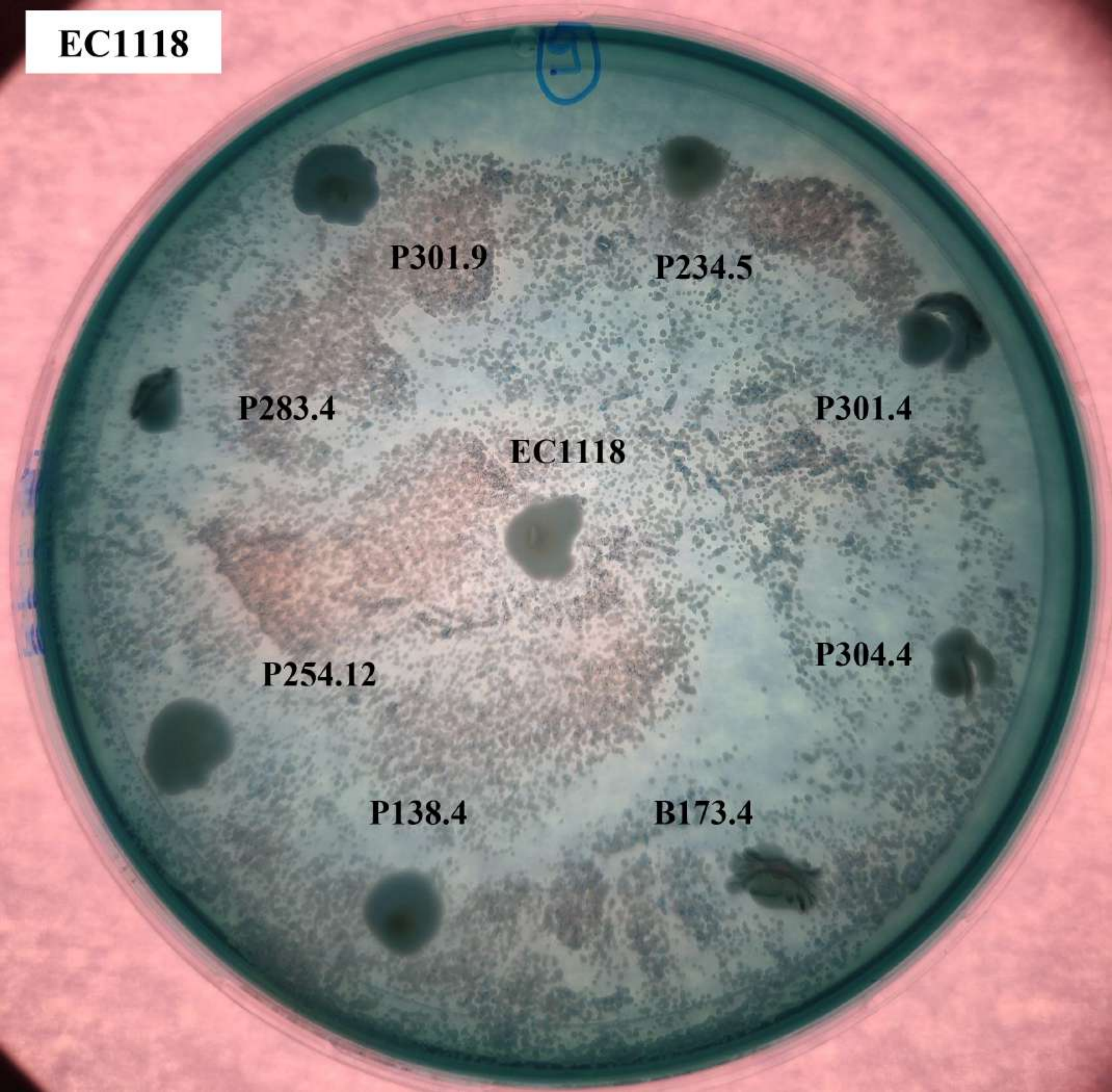
EC1118
P301.9
P234.5
P283.4
P301.4
EC1118
P254.12
P304.4
P138.4
B173.4
