# Supplementary material for: Strain prevalence and killer factor only partially influence the fermentation activity of pairwise Saccharomyces cerevisiae wine strains inoculation
Source: PLoS One. 2024 Apr 29;19(4):e0300212. doi: 10.1371/journal.pone.0300212 (PMC11057759; doi:10.1371/journal.pone.0300212)
Supplement: S1 Table — (DOCX) [file pone.0300212.s004.docx]

| **Strain** | **Glucose (g/l)** | **Glycerol (g/l)** | **Succinic acid (g/l)** | **Acetic acid (g/l)** |
| --- | --- | --- | --- | --- |
| **B173.4** | 0.70 ± 0.57 | 5.27 ± 0.08 | 0.37 ± 0.04 | 0.47 ± 0.05 |
| **P138.4** | 0.96 ± 1.17 | 4.74 ± 0.03 | 0.27 ± 0.04 | 0.38 ± 0.04 |
| **P234.15** | 0.58 ± 0.36 | 5.45 ± 0.33 | 0.38 ± 0.04 | 0.69 ± 0.16 |
| **P254.12** | 10.56 ± 2.20 | 4.70 ± 0.13 | 0.33 ± 0.01 | 0.61 ± 0.05 |
| **P283.4** | 11.46 ± 3.52 | 4.66 ± 0.12 | 0.19 ± 0.03 | 0.56 ± 0.05 |
| **P301.4** | 1.64 ± 0.78 | 4.14 ± 0.06 | 0.25 ± 0.01 | 0.40 ± 0.03 |
| **P301.9** | 1.83 ± 1.50 | 4.73 ± 0.17 | 0.32 ± 0.04 | 0.48 ± 0.07 |
| **P304.4** | 8.14 ± 5.25 | 4.13 ± 0.14 | 0.26 ± 0.05 | 0.49 ± 0.03 |
