# Supplementary material for: Strain prevalence and killer factor only partially influence the fermentation activity of pairwise Saccharomyces cerevisiae wine strains inoculation
Source: PLoS One. 2024 Apr 29;19(4):e0300212. doi: 10.1371/journal.pone.0300212 (PMC11057759; doi:10.1371/journal.pone.0300212)
Supplement: S2 Table — (DOCX) [file pone.0300212.s005.docx]

| **Strains** | **Glucose (g/l)** | **Glycerol (g/l)** | **Succinic acid (g/l)** | **Acetic acid (g/l)** |
| --- | --- | --- | --- | --- |
| **P301.4 - P254.12** | 3.94 ± 1.20 | 4.94 ± 0.15 | 0.26 ± 0.09 | 0.58 ± 0.03 |
| **P301.9/P301.4** | 3.27 ± 2.00 | 4.33 ± 0.27 | 0.19 ± 0.03 | 0.44 ± 0.10 |
| **P301.9/P254.12** | 3.46 ± 1.48 | 4.83 ± 0.26 | 0.30 ± 0.01 | 0.53 ± 0.06 |
| **P283.4/P304.4** | 24.56 ± 4.46 | 3.67 ± 0.37 | 0.24 ± 0.12 | 0.41 ± 0.04 |
| **B173.4/P254.12** | 3.71 ± 1.29 | 5.16 ± 0.12 | 0.36 ± 0.04 | 0.54 ± 0.05 |
| **P283.4/P254.12** | 6.03 ± 3.53 | 4.75 ± 0.16 | 0.27 ± 0.03 | 0.53 ± 0.07 |
| **P304.4/P254.12** | 11.47 ± 7.51 | 4.18 ± 0.05 | 0.30 ± 0.04 | 0.55 ± 0.03 |
| **P138.4/P254.12** | 2.14 ± 2.03 | 4.88 ± 0.11 | 0.33 ± 0.04 | 0.64 ± 0.05 |
| **P234.15/P254.12** | 0.24 ± 0.08 | 5.30 ± 0.17 | 0.33 ± 0.03 | 0.63 ± 0.08 |
| **P283.4/P138.4** | 2.82 ± 1.08 | 4.55 ± 0.30 | 0.33 ± 0.02 | 0.53 ± 0.05 |
| **P234.15/P138.4** | 0.42 ± 0.13 | 5.14 ± 0.17 | 0.32 ± 0.04 | 0.55 ± 0.13 |
| **B173.4/P138.4** | 4.67 ± 3.48 | 4.68 ± 0.35 | 0.33 ± 0.07 | 0.40 ± 0.03 |
| **P304.4/P138.4** | 5.67 ± 3.29 | 3.99 ± 0.19 | 0.27 ± 0.04 | 0.38 ± 0.02 |
| **P234.15/P301.9** | 0.22 ± 0.13 | 5.07 ± 0.45 | 0.33 ± 0.04 | 0.46 ± 0.18 |
| **P138.4/P301.9** | 1.09 ± 1.45 | 4.49 ± 0.24 | 0.24 ± 0.02 | 0.38 ± 0.07 |
| **B173.4/P301.9** | 1.91 ± 0.71 | 5.10 ± 0.14 | 0.32 ± 0.11 | 0.45 ± 0.16 |
| **P283.4/P301.9** | 2.72 ± 1.79 | 4.37 ± 0.33 | 0.23 ± 0.07 | 0.34 ± 0.07 |
| **P304.4/P301.9** | 13.45 ± 4.69 | 4.49 ± 0.05 | 0.24 ± 0.06 | 0.59 ± 0.06 |
| **P283.4/B173.4** | 2.40 ± 2.58 | 5.00 ± 0.03 | 0.23 ± 0.02 | 0.36 ± 0.03 |
| **P304.4/B173.4** | 5.15 ± 1.86 | 4.46 ± 0.30 | 0.18 ± 0.03 | 0.45 ± 0.13 |
| **P283.4/P234.15** | 9.46 ± 3.45 | 4.74 ± 0.22 | 0.39 ± 0.03 | 0.55 ± 0.11 |
| **P304.4/P234.15** | 10.75 ± 3.36 | 4.75 ± 0.13 | 0.25 ± 0.04 | 0.50 ± 0.07 |
| **B173.4/P234.15** | 2.95 ± 1.52 | 5.69 ± 0.23 | 0.43 ± 0.02 | 0.76 ± 0.08 |
| **P234.15/P301.4** | 0.28 ± 0.05 | 4.67 ± 0.20 | 0.30 ± 0.05 | 0.47 ± 0.05 |
| **P304.4/P301.4** | 16.54 ± 10.44 | 4.08 ± 0.12 | 0.27 ± 0.01 | 0.46 ± 0.02 |
| **P283.4/P301.4** | 4.81 ± 1.39 | 4.53 ± 0.18 | 0.26 ± 0.06 | 0.35 ± 0.02 |
| **B173.4/P301.4** | 5.36 ± 2.74 | 5.09 ± 0.16 | 0.43 ± 0.05 | 0.51 ± 0.13 |
| **P138.4/P301.4** | 0.73 ± 0.51 | 4.65 ± 0.37 | 0.28 ± 0.04 | 0.47 ± 0.11 |
