# Supplementary material for: Strain prevalence and killer factor only partially influence the fermentation activity of pairwise Saccharomyces cerevisiae wine strains inoculation
Source: PLoS One. 2024 Apr 29;19(4):e0300212. doi: 10.1371/journal.pone.0300212 (PMC11057759; doi:10.1371/journal.pone.0300212)
Supplement: S3 Table — The total number of grown colonies, the number of sampled colonies for each strain (out of a total of 12 sampled colonies) and the 95% confidence interval for the proportion are reported. Killer strains are indicated in bold and sensitive strains in italics. (DOCX) [file pone.0300212.s006.docx]

| **Strain1 - Strain 2** | **Total grown colonies** | **Sampled colonies of strain 1**  **(out of 12)** | **Estimated proportion**  **of Strain 1** |
| --- | --- | --- | --- |
| **P254.12**-*P301.4* | 33 | 11 | 79.00 % - 100% |
| *P301.4*-P304.4 | 13 | 0 | 0.00% |
| **B173.4**-P304.4 | 32 | 0 | 0.00% |
| P138.4-P304.4 | 16 | 0 | 0.00% |
| P234.15-*P301.9* | 98 | 11 | 76.95 % - 100% |
| **P283.4**-*P301.9* | 156 | 12 | 100.00% |
| **P283.4**-P304.4 | 15 | 0 | 0.00% |
| *P301.4*-*P301.9* | 51 | 0 | 0.00% |
| **P283.4**-*P301.4* | 51 | 12 | 100.00% |
| **B173.4**-*P301.4* | 48 | 12 | 100.00% |
| **P254.12**-P304.4 | 33 | 0 | 0.00% |
| **B173.4**-*P301.9* | 125 | 12 | 100.00% |
| *P301.9*-**P254.12** | 40 | 4 | 10.73% - 55.93% |
| **B173.4**-**P254.12** | 81 | 6 | 23.73% - 76.27% |
| **P283.4**-**P254.12** | 46 | 5 | 17.42% - 65.92% |
| P138.4-**P254.12** | 127 | 10 | 63.19% - 100% |
| P234.15-**P254.12** | 112 | 5 | 15.19% - 68.15% |
| **P283.4**-P138.4 | 128 | 6 | 22.96% - 77.04% |
| P234.15-P138.4 | 126 | 5 | 15.03% - 68.31% |
| **B173.4**-P138.4 | 97 | 7 | 32.08% - 84.58% |
| P138.4-*P301.9* | 113 | 7 | 31.84 % - 84.82% |
| P304.4-*P301.9* | 58 | 10 | 64.39% - 100% |
| **P283.4**-**B173.4** | 57 | 6 | 24.64% - 75.36% |
| **P283.4**-P234.15 | 35 | 7 | 35.39% - 81.27% |
| P304.4-P234.15 | 40 | 10 | 65.46% - 100% |
| **B173.4**-P234.15 | 43 | 9 | 53.95% - 96.05% |
| P234.15-*P301.4* | 42 | 7 | 34.47% - 82.19% |
| P138.4-*P301.4* | 97 | 10 | 63.49% - 100% |
